# Representation Learning of Logic Words by an RNN: From Word Sequences to Robot Actions

**DOI:** 10.3389/fnbot.2017.00070

**Published:** 2017-12-22

**Authors:** Tatsuro Yamada, Shingo Murata, Hiroaki Arie, Tetsuya Ogata

**Affiliations:** ^1^Department of Intermedia Art and Science, Waseda University, Tokyo, Japan; ^2^Department of Modern Mechanical Engineering, Waseda University, Tokyo, Japan

**Keywords:** symbol grounding, neural network, human–robot interaction, logic words, language understanding, sequence-to-sequence learning

## Abstract

An important characteristic of human language is compositionality. We can efficiently express a wide variety of real-world situations, events, and behaviors by compositionally constructing the meaning of a complex expression from a finite number of elements. Previous studies have analyzed how machine-learning models, particularly neural networks, can learn from experience to represent compositional relationships between language and robot actions with the aim of understanding the symbol grounding structure and achieving intelligent communicative agents. Such studies have mainly dealt with the words (nouns, adjectives, and verbs) that directly refer to real-world matters. In addition to these words, the current study deals with logic words, such as “not,” “and,” and “or” simultaneously. These words are not directly referring to the real world, but are logical operators that contribute to the construction of meaning in sentences. In human–robot communication, these words may be used often. The current study builds a recurrent neural network model with long short-term memory units and trains it to learn to translate sentences including logic words into robot actions. We investigate what kind of compositional representations, which mediate sentences and robot actions, emerge as the network's internal states via the learning process. Analysis after learning shows that referential words are merged with visual information and the robot's own current state, and the logical words are represented by the model in accordance with their functions as logical operators. Words such as “true,” “false,” and “not” work as non-linear transformations to encode orthogonal phrases into the same area in a memory cell state space. The word “and,” which required a robot to lift up both its hands, worked as if it was a universal quantifier. The word “or,” which required action generation that looked apparently random, was represented as an unstable space of the network's dynamical system.

## 1. Introduction

In recent years, the development of robots that work collaboratively in our living environment has attracted great attention. In many scenarios, these robots will be required to behave appropriately by understanding linguistic instruction from humans. Here, the meanings of instructions may change depending on the environment. Thus, robots must be able to flexibly adapt their behavior in accordance with the current situation or context. In the real world, no two events are identical; thus, a model that can generalize in order to translate an instruction to appropriate behavior even in novel situations is required. Specifying rules to define relations between language and behavior for various possible contexts becomes difficult and costs much more as task complexity increases. Therefore, to build a learning model that enables a robot to acquire generalizable relations from experience is especially desirable. To flexibly link language, which operates on discrete elements, to behavior, which operates within a continuous world, requires a solution to the symbol grounding problem (Harnad, 1990; Taniguchi et al., 2016).

One important characteristic of human language that enables us to describe even previously unseen situations is compositionality. In the field of formal semantics, the principle of compositionality (also referred to as Frege's principle) models a language system as follows: the meaning of a phrase or a sentence is given as a function of the meanings of its parts (e.g., words) (Partee, 2004). This principle means that the meaning of a complex expression is built from the meaning of its constituents and rules for combining them. Thanks to the compositionality of language and our cognitive ability to deal with it, humans can efficiently describe a wide variety of situations and dynamic events in the real world by compositionally constructing a complex expression from a finite number of elements. Investigating the compositional aspects of language deeply is important for understanding how human languages work in practice and for building intelligent communicative agents. Using the principle of compositionality as a base, formal semanticists attempt to build theoretical frameworks to explain the compositionality of natural language in a top-down manner.

In contrast with the top-down approach, there is a bottom-up approach that attempts to work from observation and analyze what kind of symbolic or compositional expressions emerge spontaneously through communicative tasks among humans, robots, and other intelligent agents (Steels and Kaplan, 1998; Steels and McIntyre, 1998; Steels, 2001; Kirby, 2002; Sasahara et al., 2007; Bleys et al., 2009; Schueller and Oudeyer, 2015; Spranger, 2015; Sukhbaatar et al., 2016; Wang et al., 2016; Havrylov and Titov, 2017; Lazaridou et al., 2017; Mordatch and Abbeel, 2017). In particular, in recent years, there have been many studies of multi-agent interaction, in which agents implemented with a deep learning model are developed in a mutually interactive manner and a compositional communication protocol emerges through the interaction. In Mordatch and Abbeel (2017), multiple agents situated within simulated 2D environments were given collaborative tasks in which agents had to symbolically communicate with each other to tell other agents their own goals. Before learning, symbols were meaningless. Being trained by reinforcement learning, the agents spontaneously gave the symbols shared meanings, which were sometimes interpretable by humans (e.g., “GO-TO,” “LOOK-AT”), and they became able to communicate by combining the symbols, each of which was a token representing a subject, verb, or objective. In Havrylov and Titov (2017), two long short-term memory (LSTM) networks developed their own communication protocol to express the content of images. The sender network encoded the image information as a sentence expression, and the receiver network decoded the sentence and inferred which image among alternatives was described by the sentence. The analysis showed that a natural language-like coding such as hierarchy of categories or the importance of word order could be developed.

In the bottom-up approach, there has also been much research that trained neural network models by supervised learning (Sugita and Tani, 2005; Ogata et al., 2007; Sugita and Tani, 2008; Arie et al., 2010; Tuci et al., 2011; Chuang et al., 2012; Stramandinoli et al., 2012; Ogata and Okuno, 2013; Heinrich and Wermter, 2014; Heinrich et al., 2015; Hinaut et al., 2014; Yamada et al., 2015, 2016; Zhong et al., 2017). In these studies, the example sets of language and corresponding behavior were designed and prepared by humans in advance. These sets were used as ground truth during training, and after that, compositional representations intermediating between language and behavior were self-organized in their models. For example, Sugita and Tani (2005) and Arie et al. (2010) trained recurrent neural network (RNN) models (Elman, 1990) to learn relations between 2- or 3-word sentences and corresponding robot behavior. After training, representations corresponding to verbs and nouns were topologically self-organized as different components in the feature space binding language with robot behavior. These were construed as plausible materialization of linguistic compositionality by a dynamical system approach. Tuci et al. (2011) also conducted robot experiments using a feed-forward neural network and claimed that the compositional aspects that potentially exist in the behavior space are required for embedding robot behavior into compositional semantics via language. Heinrich et al. (2015) trained an RNN model to translate a robot's visual input into a corresponding sentence at the phoneme level. After training, the activated internal states of the RNN were more correlated with the type of word (color, shape, or position) than the phonemes. Hinoshita et al. (2011) visualized a similar kind of abstract encoding by a hierarchical RNN that was activated in accordance with the categories of words, even though they trained the RNN with linguistic sequences only. Investigating such representations organized in machine learning models is valuable, not only for understanding the compositionality of language but also for building interpretable intelligent systems.

The current study follows the supervised learning approach to the integration of language and behavior. In most previous studies of this type, mainly words that are directly grounded in real-world matters have been considered. For example, nouns (e.g., ball, box) or adjectives (e.g., red, tall) correspond to characteristics of objects. Verbs (e.g., hit, push) or adverbs (e.g., quickly, slowly) correspond to characteristics of motion. However, in our language, there are more abstract words (e.g., society, justice) that are not grounded in concrete physical objects or actions. To tackle the grounding of such words, Cangelosi et al. have conducted a series of language-robot experiments from the point of view of cognitive developmental robotics (Cangelosi et al., 2010; Chuang et al., 2012; Stramandinoli et al., 2012; Zhong et al., 2014; Stramandinoli et al., 2017). In these works, a robot implemented with a neural network develops its linguistic skill step by step, beginning by acquiring relations between simple basic motions and words (e.g., “push,” “pull”) directly grounded in them and moving on to achieving relationships between more abstract actions and words (e.g., “give,” “reject”) only indirectly grounded in them through connections to basic words.

However, the current study deals with another kind of abstraction. Language expressions in this study include grounded, in other words, referential^1^ words and logic words, such as “not,” “and,” and “or”. These logical words are not directly referring to the real world but act as logical operators in the construction of the meaning of the sentence. For example, just after you have closed a door, the commands “open the door!” and “do not close the door!” can express the same behavior OPEN-DOOR^2^. In another case, the appropriate behaviors in response to “bring A or B” include BRING-A and BRING-B. These logic words have not been addressed in conventional studies of integrative learning of language and behavior. In accordance with the formulation of formal semantics, even such non-referential words working as logical operators can be handled in a unified way. In fact, in cases of actual human–robot communication, it is highly likely that these words will be used.

The current study investigates what kind of structure representing compositional relations between language and robot actions is self-organized in the space of internal states of an RNN model trained through supervised learning. Here, our designed tasks include referential words and non-referential logic words. The meanings of sentences are constructed from both word types. We analyze how logic words are processed and how their functions are represented by the RNN dynamics along with the referential words. More precisely, we apply the sequence-to-sequence learning method that has recently attracted great attention in the field of natural language processing (Sutskever et al., 2014; Bahdanau et al., 2015; Vinyals and Le, 2015; Wu et al., 2016) to the translation from sentences to robot actions and analyze representations by visualizing internal states during interactions that occur after training.

This paper is organized as follows. In section 2, we introduce the learning model. In section 3, we give the results of the learning experiment for the first task and analyze the representations acquired by the learning model in detail. In section 4, we report the results of the second task. In section 5, we discuss the results and then conclude this study.

## 2. Learning model

### 2.1. Problem formulation

The aim of the current study is to investigate how the compositional relations between language and robot actions are developed and represented internally by the model from direct experiences of interaction. Therefore, we define the interactive instruction–action task as a simple problem, learning to predict a robot's joint angles appropriate to the current situation. At each discrete time step *t* a neural network model receives a word ***w***_*t*_, visual information ***v***_*t*_, and the robot's current joint angle configuration ***j***_*t*_. An instruction sentence is given as a concatenation of some words, thus it takes some time steps. At each time step the model generates its prediction ***j***_*t*+1_ based on the input history ***w***_0 : *t*_, ***v***_0 : *t*_, and ***j***_0 : *t*_. During the instruction phase the appropriate prediction would be just keeping the current posture ***j***_*t*_. After an instruction is given, an appropriate prediction should be the generation of angles different from the current ones. An action corresponding to the instruction must also be generated as a sequence of joint angle configurations over several time steps. In our tasks, the appropriate action sequence after an instruction is determined by the combination of the instruction sequence, the visual information given simultaneously with the sentence, and the robot's current posture.

### 2.2. Model architecture and forward dynamics

**Figure 1:**
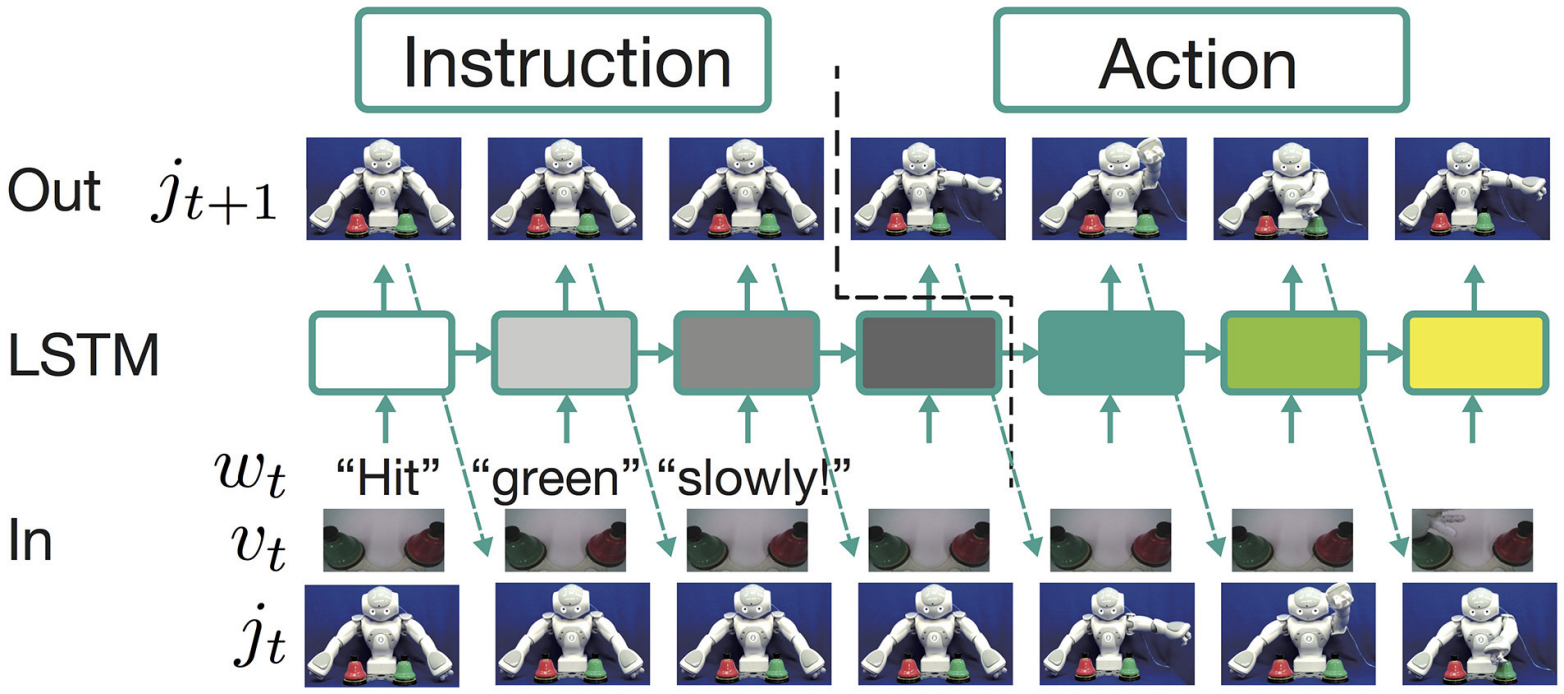
The framework employed to learn the current tasks. The learning model is a three-layer neural network whose middle layer is an LSTM layer. At each time step, the model receives word ***w***_*t*_, visual information ***v***_*t*_, and the current robot joint angles ***j***_*t*_. The LSTM layer calculates the current output ***h***_*t*_ from these external inputs, the memory cell state ***c***_*t*_, and its own output in the previous step ***h***_*t*−1_. The output layer is a fully connected layer. It receives the output of the LSTM layer and predicts the appropriate joint angles for the next time step. In this process, receiving an instruction and generating an action are completely conducted in the forward propagation algorithm. An instruction sentence, visual information, and the robot's current posture are encoded as the states of memory cells in the LSTM layer. After receiving the instruction, a corresponding action sequence is generated by decoding the integrated information.

In this study, as a model that learns the aforementioned problem, we use an RNN with an LSTM layer (Hochreiter and Schmidhuber, 1997). The model is a three-layer neural network whose middle layer is the LSTM layer, as shown in Figure 1. All the LSTM units have a peephole connection (Gers and Schmidhuber, 2000). At each time step, the model receives ***w***_*t*_, ***v***_*t*_, and ***j***_*t*_. The LSTM layer calculates the current output ***h***_*t*_ from these external inputs, the memory cell state in the previous step ***c***_*t*−1_, and its own output in the previous step ***h***_*t*−1_:

(1) $$\begin{array}{l} h_{t}=\text{LSTM}\left(w_{t},v_{t},j_{t},h_{t-1},c_{t-1};\theta \right), \end{array}$$

where **θ** denotes the parameters of the LSTM layer. In this process, ***c***_*t*−1_ is also updated to ***c***_*t*_. The output layer is a fully connected layer. It receives the output of the LSTM layer and predicts the appropriate joint angles for the next time step, denoting these ${\hat{j}}_{t+1}$. We denote the model prediction by ***j***_*t*+1_:

(2) $$\begin{array}{l} j_{t+1}=\tanh\left({Wh}_{t}+b\right), \end{array}$$

where ***W*** and ***b*** are a learnable weight matrix and a bias vector, respectively. The model prediction is also used as the joint angle input at the next time step. In this process, receiving an instruction and generating an action are completely conducted in the forward-propagation algorithm. An instruction sentence, visual information, and the robot's current posture are encoded as the states of memory cells in the LSTM layer. After receiving the instruction, a corresponding action sequence is generated by decoding the integrated information.

The working after training seems to be similar to the normal sequence-to-sequence models that have recently been used in the field of natural language processing for tasks such as question answering and translation. However, the current model is different in that it has only one LSTM layer; in other words, it does not separate the decoder from the encoder. Moreover, the algorithm does not explicitly switch between the instruction and action phases. As visually illustrated in **Figure 3** in the next section, the relations between instructions and corresponding actions are experienced entirely in the sequential data that represent human robot interaction, which consists of repeated iteration of instructions and actions. With such data, as mentioned above, the model learns to predict only the robot's joint angles appropriate for the next time step in the current situation. Because both phases are only implicitly included in the sequential data, the model has to learn to switch phases without a priori knowledge. In more precise terms, the contrasting functions of encoding and decoding (i.e., instruction receiving and action generation) emerge as an apparent phenomenon as a result of learning alone. The model continues to predict the joint angles even during receipt of an instruction, while the target is keeping the current posture. In contrast, zero-filled vectors are continuously received as language inputs even when the robot is generating an action sequence. Although no external algorithms or explicit signals on the network I/O for phase switching exist, the trained model behaves as though it flexibly switched phases. For more discussion from the point of view of dynamical systems, refer to Yamada et al. (2016).

### 2.3. Training

To train the model, supervised learning is conducted by minimizing the squared error between the model's output ***j***_*t*+1_ and the correct joint angles at the next time step ${\hat{j}}_{t+1}$: that is, the model is trained to minimize

(3) $$\begin{array}{l} E=\underset{s}{\sum }\underset{t}{\sum }{\left(j_{t+1}-{\hat{j}}_{t+1}\right)}^{2}, \end{array}$$

where *s* is the index of a sequence. The error at each time step is back-propagated to the initial time step without truncation by using the back propagation through time algorithm (Rumelhart et al., 1986). In our tasks, sometimes there are multiple correct actions. For example, if the instruction is “hit red or blue,” both HIT-RED and HIT-BLUE can be correct. In such cases, one action is chosen randomly each time and given as the correct response.

In the following sections, we describe learning experiments conducted using the model described in this section. We designed two tasks, the “flag task” and the “bell task,” in which a robot is required to generate an action in response to linguistic instructions that sometimes include logic words. Although the former task is numerically simulated on a computer from data preparation to evaluation, it is interpretable as a task for a robot. In contrast, the latter task collects motion data by using a real robot; it is, therefore, a more complicated task.

## 3. Experiment 1: flag task

### 3.1. Task overview

In this section, we first report the learning results of the first task, the “flag task”. Although this task is completely performed in a computer simulation, we describe the task as if it was undertaken by a robot so that it is easy to imagine intuitively. First, a human makes the robot grasp flags colored red, green, or blue, one in the left hand and another in the right, at random. After that, the human gives the robot a linguistic instruction. The sentence consists of a combination of an objective (“red,” “green,” “blue”), a verb [“up” (i.e., lift), “down” (i.e., lower)], and a truth value (“true,” “false”). Note that the words are given in this order because this game was designed by modifying a popular children's game in Japan. Japanese is a subject-object-verb language (cf., English, which is a subject-verb-object language), therefore a verb follows an objective word, and a truth value, which is one of the auxiliary verbs, follows a verb. Here, the objective color word indicates the arm that is grasping a flag of the stated color. The verb determines whether the flag should be raised or lowered. Finally, if the truth value is “true,” the robot must behave as indicated by the preceding verb. In contrast, if “false,” it must generate the opposite action. For example, if the robot receives an instruction “red up false” when it is grasping a red flag in the right arm, the correct action is to lower the right arm (R-DOWN). In other words, “true” and “false” roughly represent “do” and “do not,” respectively.

**Figure 2:**
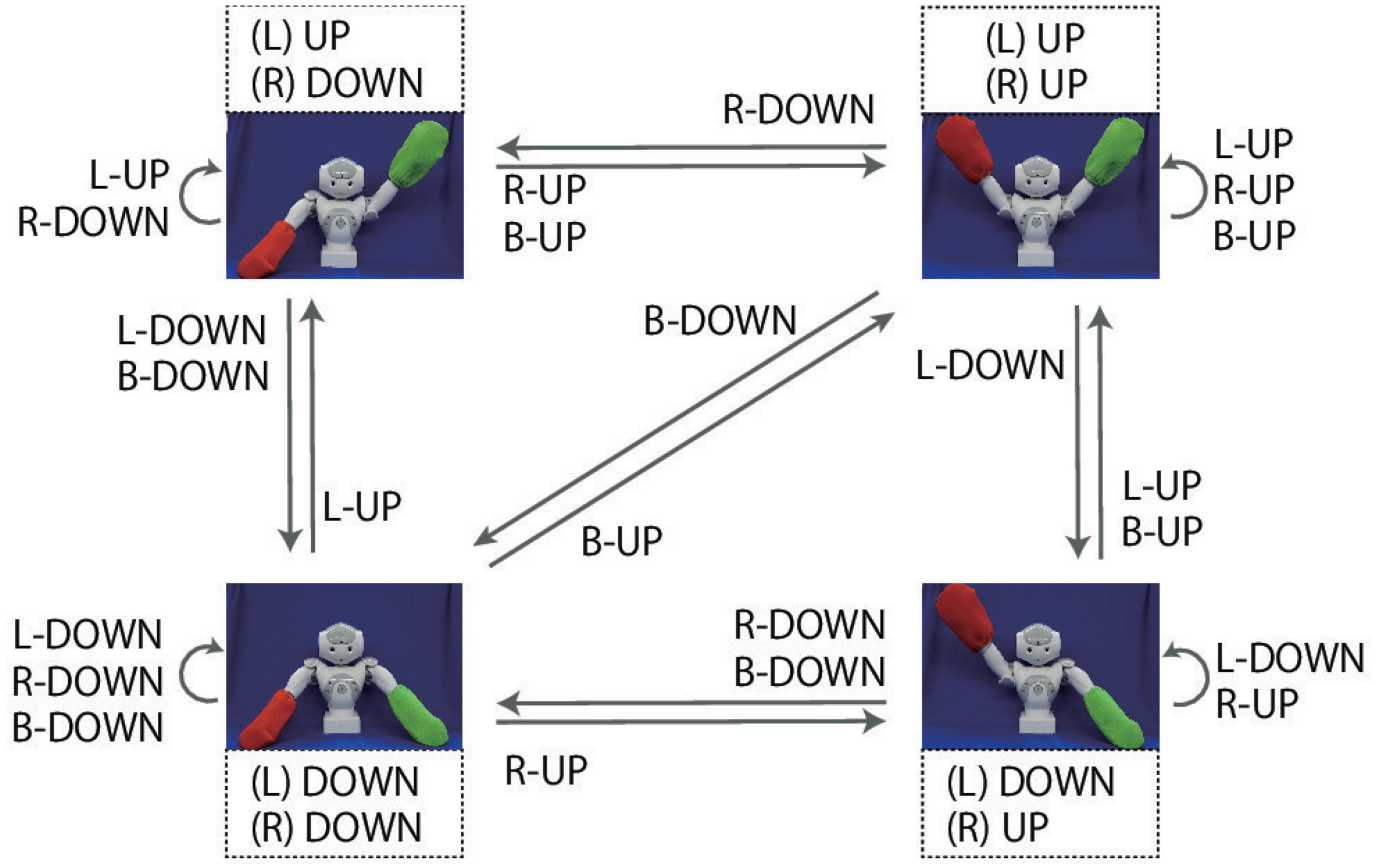
Overview of the flag task. The experimenter makes the robot grasp two colored flags. Instructions are given as sentences in the form of an objective (“red,” “green,” “blue”), a verb [“up” (i.e., lift), “down” (i.e., lower)] and a truth value (“true,” “false”). The robot must generate one of six goal-oriented actions (L-UP, L-DOWN, R-UP, R-DOWN, B-UP, B-DOWN) in accordance with the instruction. In the objective parts, two color words can be concatenated by “and”. In this case, the robot must generate B-UP (B-DOWN) action. Two color words also can be concatenated by “or,” in which case the robot must move either arm. The actual movements corresponding to these goal-oriented actions for each starting posture are indicated by the arrows in this figure.

In the objective part, two color words can be concatenated by “and” (referred to as AND-concatenated). For example, if the robot receives the instruction “red and blue up true” when it is grasping red and blue flags, the robot must lift up both arms. There are also cases in which two color words are concatenated by “or” (referred to as OR-concatenated). For example, if the robot receives the instruction “green or blue up false” when it is grasping the green and blue flags, the correct action is to lower either arm. However, if at least one arm is already in the DOWN posture, the robot must keep the current posture. The number of possible goal-oriented actions is six: L-UP, R-UP, B-UP, L-DOWN, R-DOWN, and B-DOWN, where L, R, and B mean left, right, and both, respectively. However, there are situations in which, even though the same goal-oriented action is required, the actual motion that should be generated by the robot varies according to the robot's current posture (shown as arrows in Figure 2). Note that there are even cases in which the robot should not move either of its arms. The number of possible situations, based on the combination of flag colors (6 patterns), instructions (24 patterns), and the robot's waiting posture (4 patterns), is 576. In this task, instructions inconsistent with the flag colors are never given. For example, if the colors of the flags held by the robot are red and blue, the instruction “green up true” is never given. Furthermore, cases in which both flags are the same color are not permitted.

The requirements imposed on the robot in this game are analyzed as follows. (1) First, the arm indicated by the color words depends on the arm with which the robot holds the flag. In other words, referring to an external situation is required. (2) The actual motion trajectory to be generated depends on the robot's current posture. For example, suppose the robot is required to generate L-UP action. If the robot's left arm is in the DOWN posture, the robot has to lift its left arm. However, if the robot's left arm is already in the UP posture, the robot has to maintain its posture. (3) Finally, the RNN has to deal not only with referential words (e.g., verb, objective) but also logic words such as “true,” “false,” “and,” and “or,” which we focus on in the current study. Due to this task setting, in extreme cases, sentences completely orthogonal to each other can indicate the same action (e.g., “red up true” with the red flag in the left arm and “blue down false” with the blue flag in the left arm). In contrast, some OR-concatenated sentences have an ambiguity that allows the robot multiple choices even in the same situation.

### 3.2. Data representation

We represent the execution of the flag task as a sequence of 14-dimensional vectors. The state ***S***_*t*_ at time step *t* is represented as follows:

(4) $$\begin{array}{l} j_{t}=\left[j_{l}^{\left(t\right)},j_{r}^{\left(t\right)}\right], \end{array}$$

(5) $$\begin{array}{l} v_{t}=\left[v_{r}^{\left(t\right)},v_{g}^{\left(t\right)},v_{b}^{\left(t\right)}\right], \end{array}$$

(6) $$\begin{array}{l} w_{t}=\left[w_{0}^{\left(t\right)},w_{1}^{\left(t\right)},w_{2}^{\left(t\right)},w_{3}^{\left(t\right)},w_{4}^{\left(t\right)},w_{5}^{\left(t\right)},w_{6}^{\left(t\right)},w_{7}^{\left(t\right)},w_{8}^{\left(t\right)}\right], \end{array}$$

(7) $$\begin{array}{l} S_{t}=\left[j_{t};v_{t};w_{t}\right]. \end{array}$$

**Figure 3:**
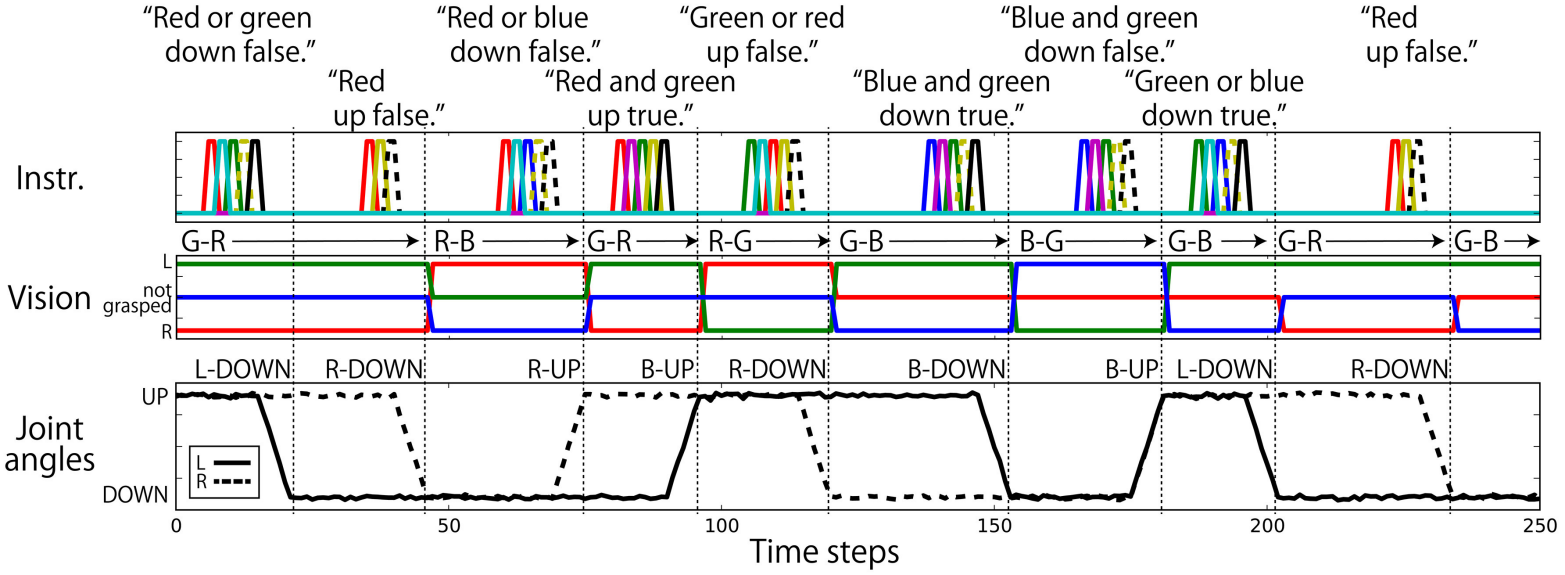
An example sequence that represents the flag task. Each vertical broken line indicates the end of an episode. **(Top)** An instruction is given as a succession of words, which are each represented as a 1-hot vector. In the waiting and action-generation phases, zero-filled vectors are given. **(Middle)** Visual information is continuously given as a sequence of three-element (R, G, B) vectors. The flag colors can be changed randomly just after action generation. Because this task was numerically simulated on a computer, changes in flags were represented as instantaneous changes in values. Note that flags are sometimes not changed as in the case from the first episode to the second episode in this figure. **(Bottom)** Each action immediately follows an instruction.

Regarding the robot joints, only the left and right shoulder pitches ($j_{l}^{\left(t\right)},j_{r}^{\left(t\right)}$) are used. The permissible range of each shoulder pitch is scaled in the interval [−1.0, 1.0]. The UP posture corresponds to a pitch of 0.8, and the DOWN posture corresponds to a pitch of −0.8. Posture changes from UP to DOWN or from DOWN to UP after receiving an instruction are completed over 6 time steps. Visual information is represented in 3 dimensions ($v_{r}^{\left(t\right)},v_{g}^{\left(t\right)},v_{b}^{\left(t\right)}$). The three components correspond to the R, G, and B channels, respectively. If the color is grasped by the left hand, the component is set to 0.8; if it is in the right hand, the component is −0.8; and if not grasped by either hand, the component is 0.0. Nine elements are assigned for language. Each element corresponds to one word, out of “red,” “green,” “blue,” “up,” “down,” “true,” “false,” “and,” and “or,” and an instruction sentence is represented as a sequence of one-hot vectors, which have the value of 0.8 at one element and 0.0 at the other element. In this study, the data representing the flag task are completely generated on a computer without using a real robot. Example interaction data are shown in Figure 3. Note that we added a small amount of Gaussian noise (mean: 0.00; standard deviation: 0.02) to the values of joint angles. In the preliminary experiment, we first trained the model without noise and got poor results. We then added noise and the results improved. We discuss this effect in section 5.

### 3.3. Learning setting and evaluation method

We made 2,048 sequential datasets for training, each of which includes 10 episodes. The term, “episode” denotes a chunk consisting of an instruction and an action response. The situations included in each sequence were randomly ordered. All 576 possible situations were included at least once. We built five models with 50, 70, 100, 150, and 300 LSTM units and trained them 10 times from randomly initialized learnable parameters. We also trained the 100-node model with data without noise applied to the joint angles. Adam, a version of the stochastic gradient descent algorithm made stable by computing individual adaptive learning rates for each parameter, is used as an optimizer (for details, refer to Kingma and Ba, 2015). The number of learning iterations is 10,000, and the learning rate is set to 0.001. We coded our model within Python using the Chainer (https://chainer.org) framework. The source code of our model is available at https://github.com/ogata-lab/RNN_FNR2017.

After learning, we made another dataset for the evaluation. This dataset includes all the possible situations 10 times each. Although the situations were randomly ordered, the order was different from the training dataset. When the errors between the generated postures of both arms six steps after receiving an instruction and the correct ones are less than 0.04, we judge that the RNN has succeeded in generating an appropriate action. Here, there are cases in which the correct action cannot be determined uniquely. In such cases, if the RNN succeeds in generating any of the correct actions, we judge that as success. We regard the situation patterns in which the RNN succeeds in generating an appropriate action more than seven times out of 10 as “appropriately learned”. Note that in the current task, the sequences are given to the robot as multiple repetitions of the instructions and corresponding actions. Therefore, even if situations that are defined by combination of an instruction, the vision, and the robot posture are the same, slightly different activations are gained every time because the contextual information of the previous episode remains in the memory cell states. Thus, the generated action is not identical among trials.

### 3.4. Task performance after training

**Figure 4:**
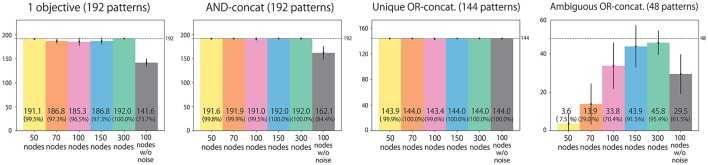
Experiment 1 (flag task). Action generation performance. We evaluated performance by counting how many situation patterns each model learned appropriately with respect to each of the four situation types: (1) the instruction includes one objective; (2) the instruction is AND-concatenated; (3) the instruction is OR-concatenated, but there is only one correct action; and (4) the instruction is OR-concatenated, and two correct actions exist. Note that the written values are averages of 10 trials in which learning began with different seeds. Error bars represent standard deviations.

**Figure 5:**
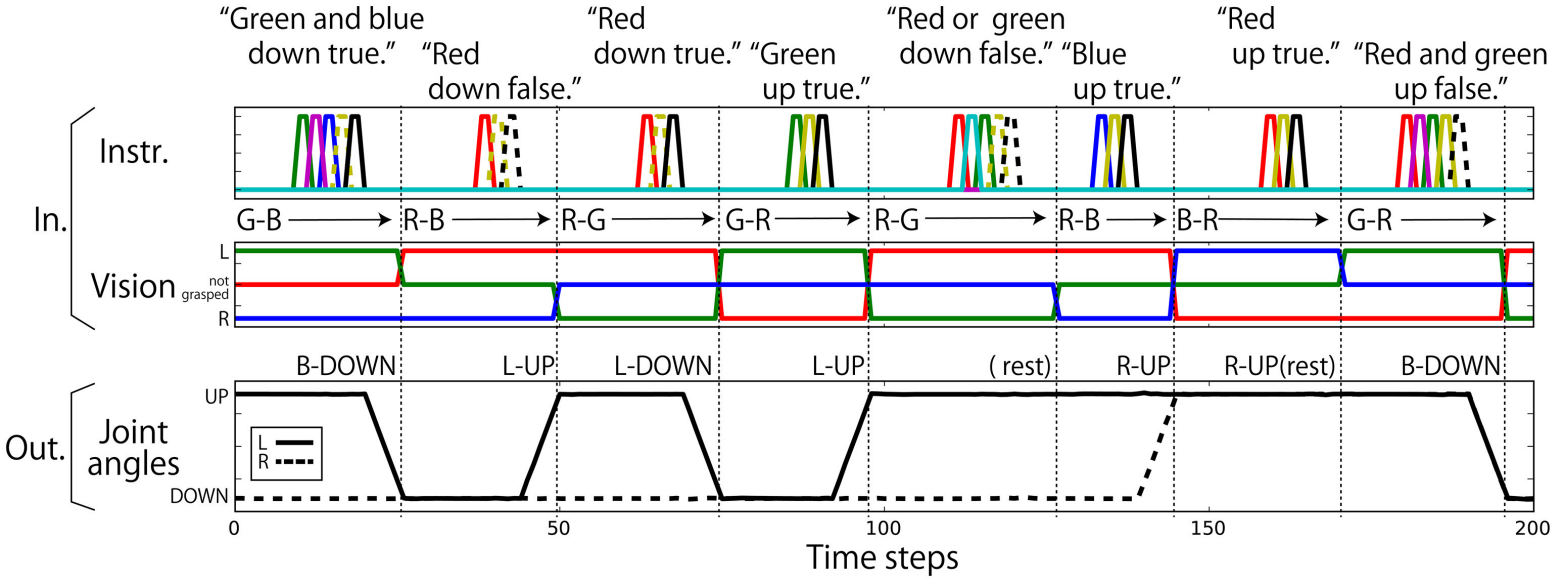
An example of the resulting interaction in the flag task. The 300-node model could generate an appropriate action in almost all situations.

We classify all possible situations into four types. (1) Situations in which the instruction includes only one objective word (192 situations). (2) Situations in which the instruction is AND-concatenated (192 situations). (3) Situations in which the instruction is OR-concatenated, but there is only one correct action. For example, when the instruction is “red or blue up true” and the both arms are already in the UP position, the only correct action is to maintain the UP-UP posture (144 situations). (4) Situations in which the instruction is OR-concatenated, and two correct actions exist (48 situations). We evaluate performance by counting how many situation patterns each model learns appropriately with respect to each of the four types. Figure 4 shows the result. Most situations in types (1), (2), and (3), in which the correct action is uniquely determined, were appropriately learned by all the models. However, the 100-node model trained with data without noise applied to the joints could not learn sufficiently well. For type (4), in which the correct action cannot be determined uniquely, a clear difference exists between models: the number of appropriately learned situations increased in accordance with the number of LSTM nodes. The model without noise also performed worse than the 100-node model with noise. Figure 5 shows an actual example of interaction achieved by the 300-node model. It can be seen that the RNN generates an appropriate action immediately after receiving an instruction in each episode.

Next, we checked which arm was actually moved in situations of type (4). If the model learned the type (4) situations just as a left-arm action or just as a right-arm action, the meaning of “or” cannot be regarded as being truly learned, although the aforementioned evaluation criteria is fulfilled. Here, we investigated the results for a model with 300 LSTM units. In 45.4% of the trials, the left hand was moved. In 52.5%, the right hand was moved. In 2.1%, neither movement could be generated successfully. Overall, the arms were quite evenly chosen in these situations. There are 48 situation patterns of type (4), and the test was conducted 10 times for each of them. In all cases, the RNN sometimes chose to move the left arm and other times chose to move the right arm. In other words, the RNN could learn the meaning of OR-concatenated instructions appropriately as “OR”. Thus, the flag task was performed sufficiently well by the trained models.

### 3.5. Analyses of internal representations

In the previous subsection, we confirmed that the RNN could learn to execute the flag task. In this section, to analyze how the RNN internally represents the relations between instructions and sensorimotor information, we visualized the internal states during the execution of the task by principal component analysis (PCA)^3^.

#### 3.5.1. Representations of referential color words

**Figure 6:**
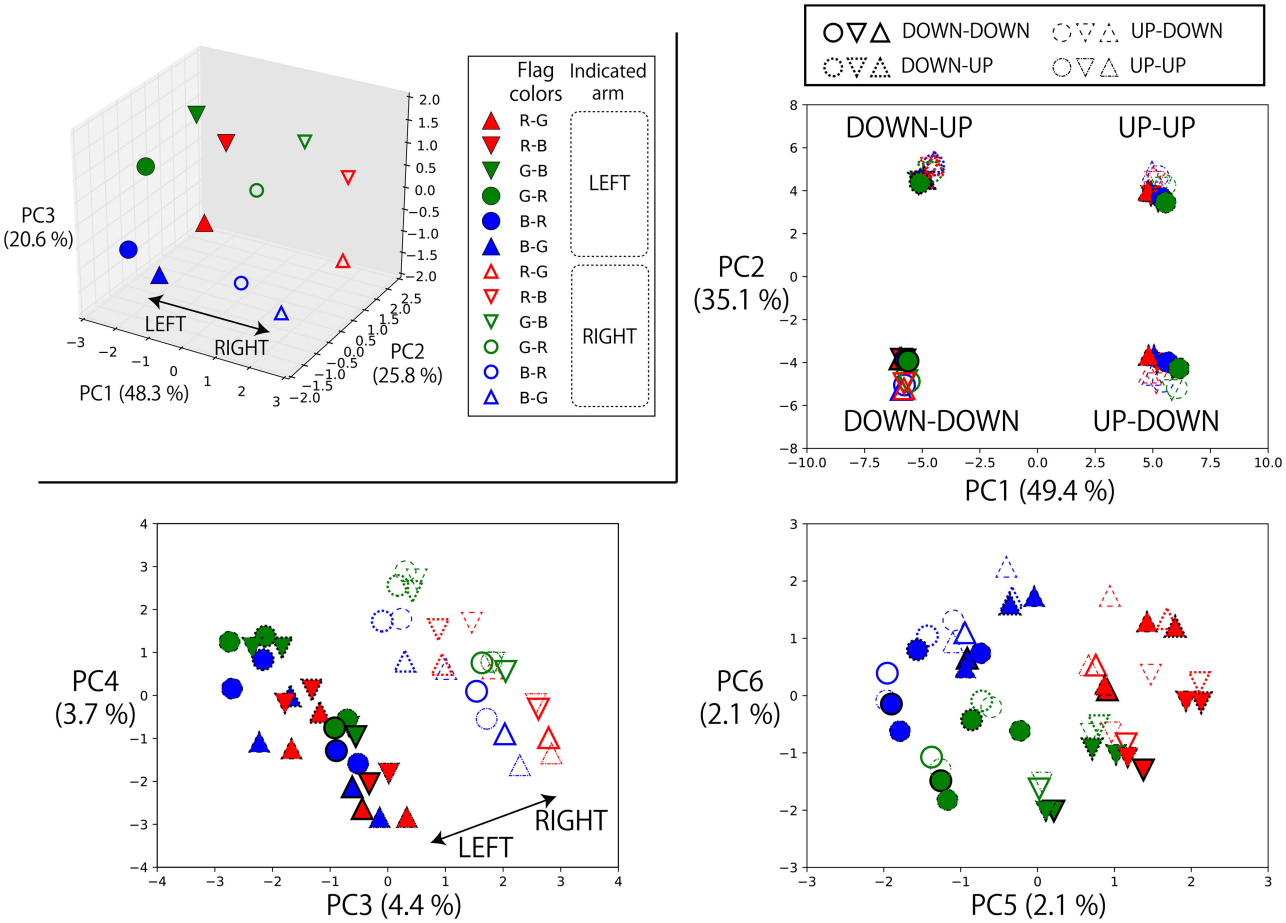
Top left: The states of the memory cells after the instruction “(L-flag color word) up true” or “(R-flag color word) up true” is given to the robot projected onto the space spanned by PC1, 2, and 3. Here, the robot is always waiting in the DOWN-DOWN posture, but the situations are different with respect to the colors of the flags grasped in each hand. For example, the filled blue circle is the activation after receiving “blue up true” in the situation B-R in which a blue flag is in the left hand and a red flag is in the right. In this task, which arm should be moved cannot be determined from the given objective word alone. However, in the PC1 direction, which arm is indicated by the objective word is represented. The RNN learned to integrate the objective word information and the current visual information, and acquired a representation corresponding to the meaningful pair of “left–right”. By using these activations, the robot could choose a correct arm for each trial. Others: We also plotted the internal states after giving these instructions to the robot that is waiting in the other postures, together with the internal states on the DOWN-DOWN condition. We projected them onto the PC1–2, PC3–4, and PC5–6 space. Note that we carried out PCA again by using the internal states on all of these conditions. Plot colors and shapes are as in the top left panel except that the frame lines differ according to the robot current posture. In this case, the current posture information is strongly reflected to the internal states, thus it is encoded in the PC1–2 plane. But the representation corresponding to “left” and “right” is still able to be seen easily in the PC3–4 plane. The visual information was encoded in the PC5–6 space although the hexagon shape was a little distorted.

First, the top left panel of Figure 6 shows the states of the memory cells after the instruction “(L-flag color word) up true” or “(R-flag color word) up true” is given to the robot. Here, the robot is always waiting in the DOWN-DOWN posture, but the situations are different with respect to the flag colors. Therefore, the RNN has to choose which arm should be raised by integrating the visual information and the input objective word. In the PC2–PC3 plane, the current visual input is directly embedded. However, in the PC1 direction, which arm has been indicated by an objective word is represented. In other words, in the experience of generating action sequences by receiving an instruction and visual input, the RNN acquired a representation corresponding to the meaningful pair of “left” and “right”. We also plotted the internal states after giving these instructions to the robot that is waiting in the other postures, together with the internal states on the DOWN-DOWN condition. In the other three panels of Figure 6, we projected them onto the PC1–2, PC3–4, and PC5–6 space. In this case, the current posture information is strongly reflected to the internal states, thus it is encoded in the PC1–2 plane. But the representation corresponding to “left” and “right” is still able to be seen easily in the PC3–4 plane. Here, note that in the case of the UP-UP posture, the actual motions to be generated by receiving “(L-flag color word) up true” or “(R-flag color word) up true” are the same (keep the current posture), and, in fact, the network could keep the posture. This analysis shows that even in such situations in which the same action was generated, the model could internally represent these instructions as different meanings, “left” or “right”. Incidentally, the visual information was also still encoded in a less principal component space (PC5–6) although the hexagon shape was a little distorted.

#### 3.5.2. Representations of logic words: “true” and “false”

**Figure 7:**
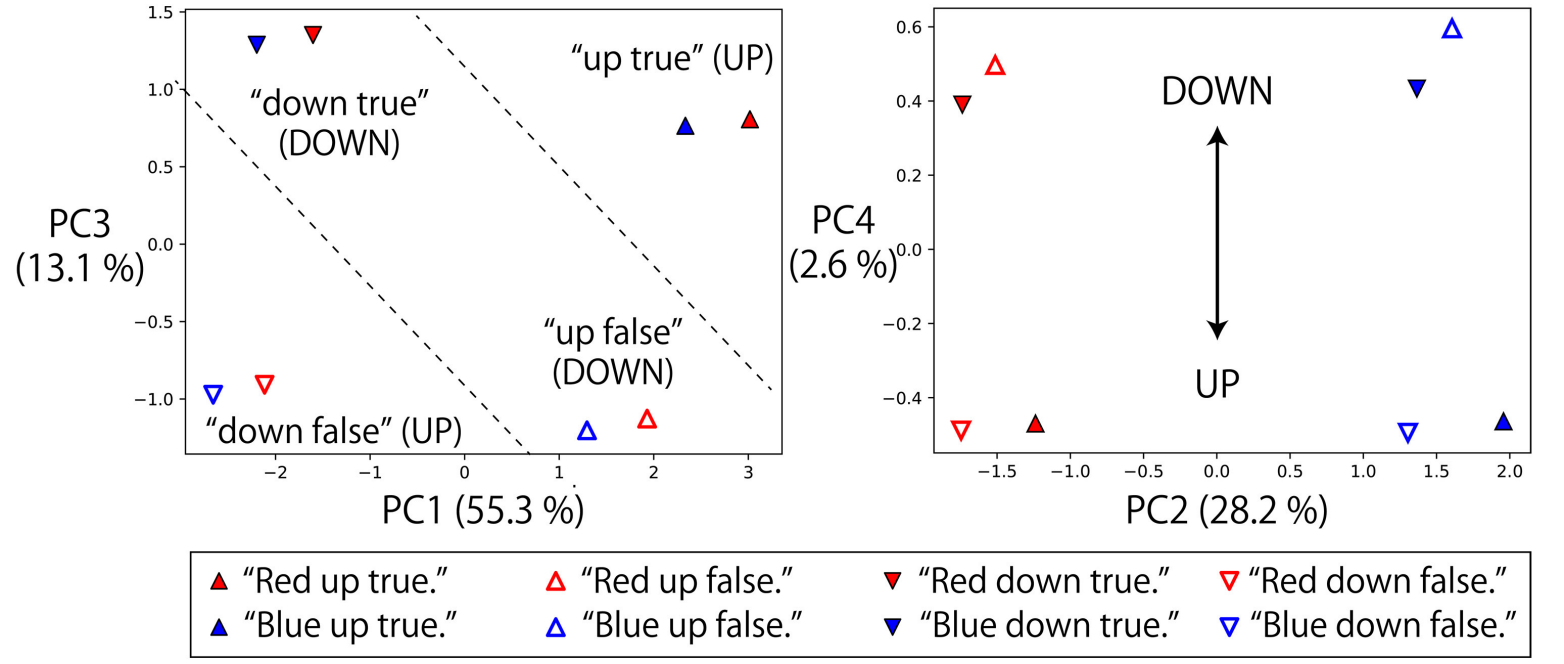
Memory cell states after giving eight possible instructions with one objective word to the RNN in the situation that the grasped flags are R-B and the waiting posture is DOWN-DOWN. The left panel projects them onto PC1–3 space, and the right panel projects them onto PC2–4 space. In the directions of PC1, PC2, and PC3, the activations directly corresponding to each part of speech (objective, verb, truth value) can be seen: that is, “red”/“blue”, “up”/“down” and “true”/“false” pairs are reflected in the PC2, PC1, and PC3 axes, respectively. However, by exploring lower rank components, it can be seen that the X-OR problem consisting of “up”/“down” and “true”/“false” pairs is solved in the PC4 direction by non-linearly embedding the input sentences.

Next, we also analyzed the representations of logic words. We visualized memory cell activations after giving eight possible instructions with one objective word to a robot that was grasping R-B flags and waiting in the DOWN-DOWN posture (Figure 7). In the directions of PC1, PC2, and PC3, the activations directly corresponding to each part of speech (objective, verb, truth value) of the input sentence can be seen, that is, “red”/“blue”, “up”/“down” and “true”/“false” pairs are reflected in the PC2, PC1, and PC3 axes, respectively. Here, the problem is that the RNN has to solve an X-OR problem that consists of “up”/“down” and “true”/“false” (shown in the left panel of Figure 7), and to link its interpretation into UP or DOWN goal-oriented action. More precisely, if the sentence includes “up true” or “down false,” UP action must be chosen. In contrast, if the sentence includes “up false” or “down true,” DOWN action must be chosen.

Actually, by exploring the lower-rank component PC4, the activations that were located diagonally across the parallelogram in PC1–PC3 space were located in the same direction. “Up true” and “down false,” which are mutually orthogonal but have the same meaning UP, are represented in the bottom area of the right panel. In contrast, “up false” and “down true” are represented in the top area. Thanks to this non-linear embedding, the X-OR problem is solved in the PC4 direction. In summary, the RNN has extracted the XOR problem implicitly included in the sequential experiences and learned to link the orthogonal instructions in the same goal-oriented action by its non-linear dynamics, while retaining the information that the input sentences are very different from each other in the larger principal components.

#### 3.5.3. Representations of logic words: “and” and “or”

**Figure 8:**
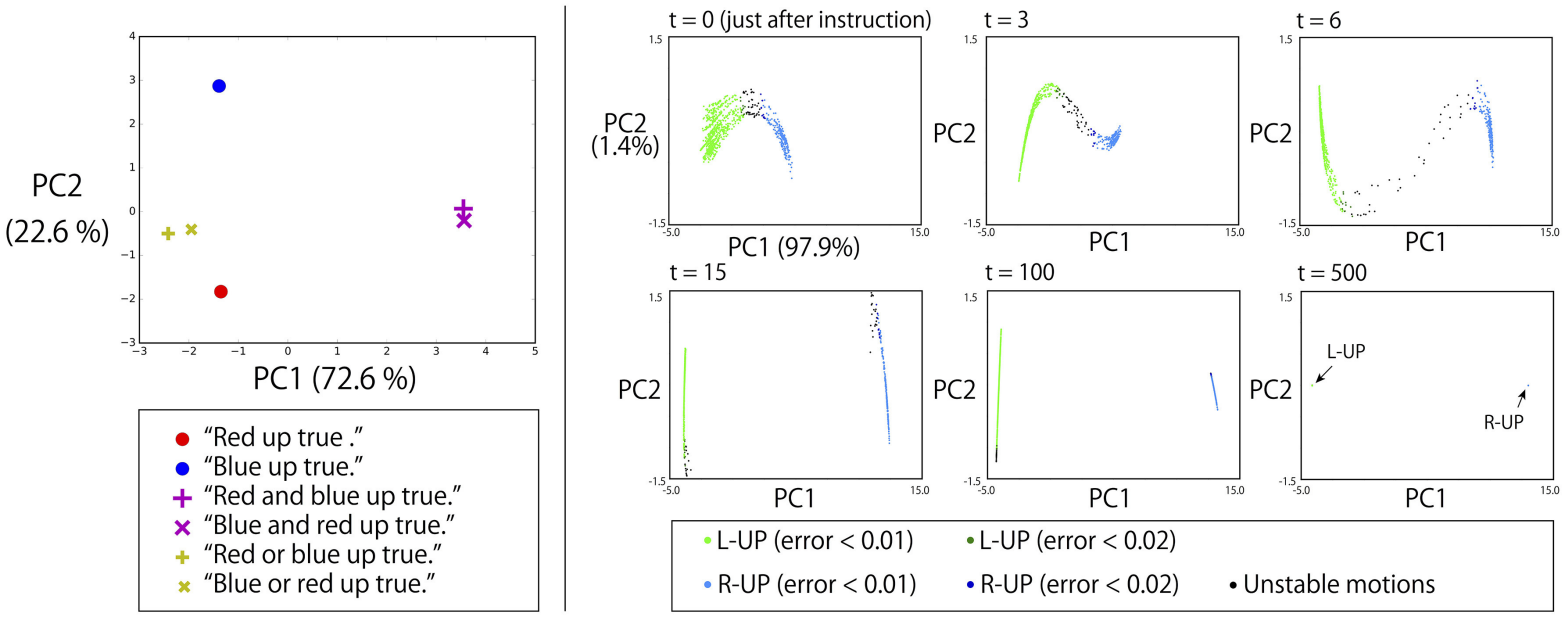
**Left**: The memory cell states after giving a robot that is grasping red and blue flags some instructions whose objective part is one word, AND-concatenated, or OR-concatenated. The verb and the truth value are “up” and “true,” respectively. The AND-concatenated instructions are represented away from other instruction encodings in the PC1 direction. The pair of “red” and “blue” is represented in the PC2 direction. The “or” that directs the robot to raise either hand is embedded in the middle space between these two encodings. **Right**: To a robot waiting in the DOWN-DOWN posture with G-B flags after 2,048 different contexts, we gave the instruction “green or blue up true.” The memory cell states after the instruction (*t* = 0) were arranged on an arch-shaped space **(left top)**. Each point corresponds to one specific context. When the activation was on the left side of the arch, the robot generated L-UP action and the internal states converged to the fixed-point corresponding to the UP-DOWN posture. In contrast, on the right side, the robot generated R-UP action, and the internal states converged to the fixed-point corresponding to the DOWN-UP posture. When the activation was on the topmost area of the arch, a little unstable action was generated. However, even in such cases, the internal states eventually converged to one of fixed-points, as shown in the right bottom panel.

The left panel of Figure 8 shows the memory cell states after giving a robot that is grasping R-B flags some instructions whose objective part is one word, AND-concatenated, or OR-concatenated. The verb and the truth value are “up” and “true,” respectively. AND-concatenated instructions that direct the robot to raise both arms are represented away from other instruction encodings in the PC1 direction. The pair of “red” and “blue” is represented in the PC2 direction. Here, the word “or” that directs the robot to raise either hand is embedded in the middle space between these two encodings. This suggests that “or” is represented as an unstable point of the network dynamics and that, thanks to this acquired dynamics, behavior which apparently looks like randomly choosing the left or right arm has emerged.

To verify this, we conducted the following additional simulation. To a robot that had 2,048 different contexts, we gave the instruction “green or blue up true.” Specifically, in all 2,048 contexts, a robot is currently waiting in a DOWN-DOWN posture with G-B flags. However, in each context, the order of preceding episodes is randomly different from in the other contexts. As mentioned in section 3.3, even when the situation, defined by the combination of an instruction, the vision, and the robot current posture (in this simulation, “green or blue up true,” the green flag in the left hand, the blue flag in the right hand, and DOWN-DOWN posture, respectively) is the same, different activations occur every time because the contextual information of the previous episodes still remains in the memory cell states. Therefore, we see 2,048 different activations corresponding to 2,048 contexts. As shown in the top left panel of the right side of Figure 8, the memory cell states after the instruction “green or blue up true” were then arranged in an arch-shaped space. Each point corresponds to one specific context. When the activation was on the left side of the arch, the robot generated L-UP action. In contrast, for right-side activation, the robot generated R-UP action. When the activation was in the topmost area of the arch, some unstable motion was generated. However, in all cases, the internal states eventually converged into one of the fixed-point attractors that corresponded to the DOWN-UP posture or the UP-DOWN posture, as shown in the bottom rightmost panel of Figure 8. This means that to respond to OR instructions that require the robot to behave in a random exclusive-OR-like way, the internal representation was the convergence from an unstable space to either one of two stable points.

In this analysis, PC1 was strongly dominant (the contribution ratio is 97.9%). Therefore, due to this important contribution ratio, one could assume that only one neuron would be enough to generate this unstable dynamics. However, the activation in the PC1 direction was actually composed of the activations of multiple units. Specifically, no single unit has cosine similarity of more than 0.4 (or less than −0.4) to PC1. Instead, seven units have cosine similarity of in the range between 0.2 and 0.4 (or between −0.4 and −0.2) to PC1. In other words, this unstable dynamics was realized in a distributed way.

#### 3.5.4. Dynamical representations of the task execution

**Figure 9:**
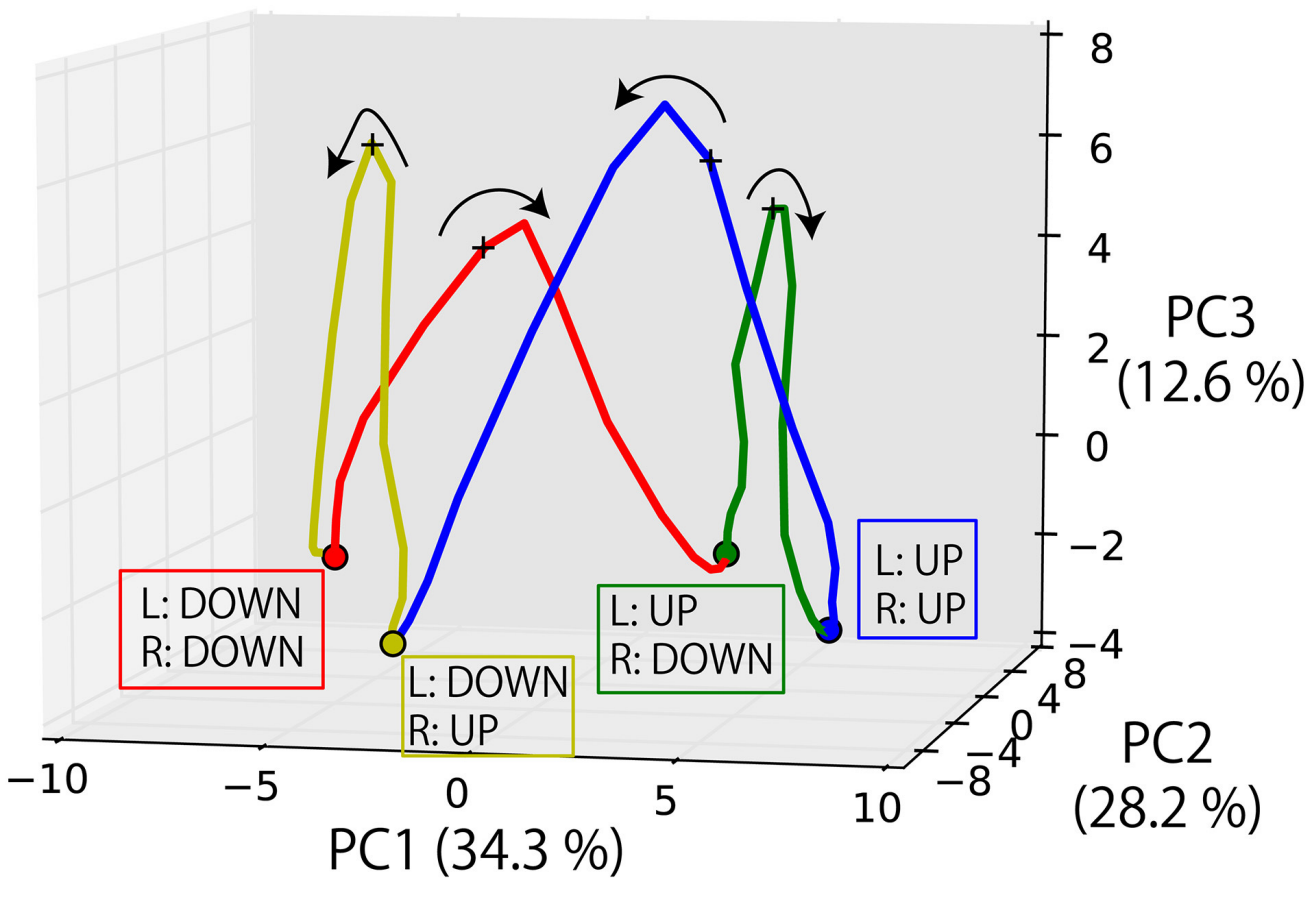
The state transition of memory cells while the robot experienced four episodes and its posture moved in the order from DOWN-DOWN, through UP-DOWN, UP-UP, DOWN-UP, to DOWN-DOWN. The transitions among different postures are represented as transitions among different fixed-point attractors (circle marks), each of which corresponds to a posture. By receiving an instruction, the internal state is activated in the PC3 direction and reaches the unstable point indicated by a + mark. By converging into one of the fixed-points again after the activation, the corresponding goal-oriented action is generated. The robot then waits for a subsequent instruction at that point.

Finally, we visualized the internal dynamics during the execution of the task. Figure 9 shows the state transition of memory cells while the robot experienced four episodes and its posture is moved in the order from DOWN-DOWN, through UP-DOWN, UP-UP, DOWN-UP, to DOWN-DOWN. Here, the PC1-2 space seems to roughly correspond to the robot's posture. Moreover, the transitions among different postures are represented as transitions among different fixed-point attractors (shown as circles), each of which corresponds to a posture. By receiving an instruction, the internal state is activated in the PC3 direction and reaches the unstable point indicated by a + mark. This activation is gained as a result of the integration of the visual information and processing logic words, as mentioned above, although it is difficult to visualize them simultaneously in this figure. By converging to one of the fixed-points again after the activation, the corresponding goal-oriented action is generated. The robot then waits for a subsequent instruction at that point. This is the case even when the correct action is to maintain the current posture. While the apparent motion of joint angles is remaining stationary, it was internally represented as converging to the original fixed-point.

In summary, the RNN learned to encode the instructions in a form integrated with the visual inputs and the current robot posture and to generate an appropriate robot action through the experience of sequential interaction data. It was also revealed that logical words, “true,” “false,” “and,” “or” are processed along with the other referential words and encoded in a way that reflects the functions in the current task.

### 3.6. Generalization ability

**Figure 10:**
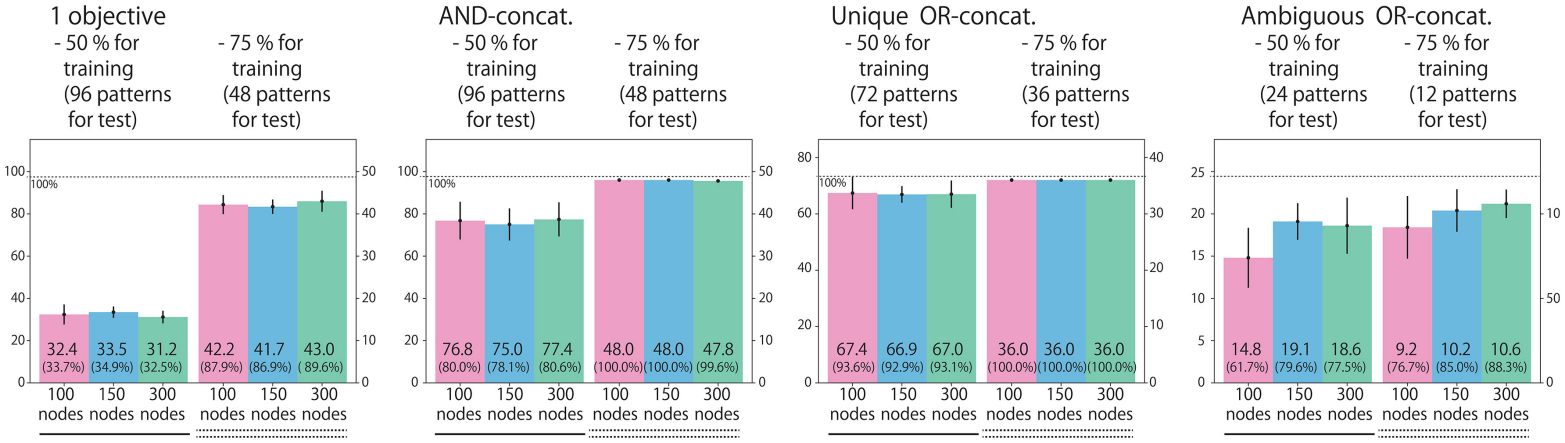
To evaluate the model's generalization ability for the flag task, we conducted learning experiments again by removing (a) 50% or (b) 25% of the possible situations from training dataset. We evaluated the performance by counting how many unexperienced situation patterns each model dealt with appropriately. Similarly to Figure 4, we evaluated the performances with respect to each of the four situation types.

In the previous subsection, we showed the internal representations of relations between instructions and actions acquired through the experience of an imposed task. Empirically, when such kinds of systematic representation can be organized, the model achieves a certain level of generalization ability (Sugita and Tani, 2005; Ogata et al., 2007; Yamada et al., 2016). Thus, we conducted learning experiments again by removing 50 or 25% of the possible situations from the training dataset. We chose removed patterns regularly so that each word, robot posture, and flag arrangement would appear uniformly, as shown in Table 1. Here, we trained only three models with 100, 150, and 300 LSTM units. The results are shown in Figure 10.

**Table 1:** To evaluate the model's generalization ability for the flag task, we conducted learning experiments again by removing (a) 50% or (b) 25% of the possible situations from training dataset.

**Posture**	**Colors**	**Instructions**															
		**LUT**	**LUF**	**LDT**	**LDF**	**RUT**	**RUF**	**RDT**	**RDF**	**AUT**	**AUF**	**ADT**	**ADF**	**OUT**	**OUF**	**ODT**	**ODF**
DOWN-DOWN	R-G	◦	⊚		⊚	⊚		⊚	◦	◦		⊚	⊚	⊚		⊚	◦
	G-R	⊚		⊚	◦		⊚	◦	⊚	⊚	◦	⊚			⊚	◦	⊚
	G-B	◦	⊚	⊚		◦	⊚	⊚			⊚	◦	⊚	⊚	⊚		◦
	B-G	⊚	◦		⊚	⊚	◦		⊚	◦	⊚	⊚		◦		⊚	⊚
	B-R		◦	⊚	⊚	⊚	⊚		◦	⊚	◦		⊚	⊚	◦	⊚	
	R-B	⊚	⊚	◦		◦		⊚	⊚	⊚	⊚		◦		⊚	◦	⊚
DOWN-UP	R-G		◦	⊚	⊚	⊚	⊚	◦		⊚		◦	⊚	⊚	◦	⊚	
	G-R	⊚	⊚		◦		◦	⊚	⊚	⊚	⊚		◦	◦		⊚	⊚
	G-B	◦	⊚		⊚	⊚		⊚	◦	◦		⊚	⊚	⊚	⊚		◦
	B-G	⊚	◦	⊚		◦	⊚		⊚	⊚	◦	⊚		⊚	◦		⊚
	B-R		⊚	⊚	◦		⊚	⊚	◦		⊚	◦	⊚		⊚	⊚	◦
	R-B	⊚		◦	⊚	⊚	◦		⊚		⊚	⊚	◦	◦	⊚		⊚
UP-DOWN	R-G	◦	⊚	⊚		◦	⊚	⊚			⊚	◦	⊚	◦	⊚	⊚	
	G-R	⊚		◦	⊚	⊚		◦	⊚	◦	⊚	⊚			⊚	◦	⊚
	G-B	◦		⊚	⊚	⊚	⊚		◦	⊚	◦		⊚	⊚		⊚	◦
	B-G	⊚	⊚		◦		◦	⊚	⊚	⊚	⊚		◦		◦	⊚	⊚
	B-R		⊚	◦	⊚	⊚	◦	⊚		◦		⊚	⊚	⊚	⊚	◦	
	R-B	⊚	◦	⊚			⊚	◦	⊚	⊚		⊚	◦	⊚	◦		⊚
UP-UP	R-G	⊚	⊚	◦		◦		⊚	⊚	⊚	⊚	◦		⊚		⊚	◦
	G-R		◦	⊚	⊚	⊚	⊚	◦		⊚	◦		⊚		⊚	◦	⊚
	G-B	⊚		◦	⊚	⊚	◦		⊚		⊚	⊚	◦	◦		⊚	⊚
	B-G		⊚	⊚	◦	◦	⊚	⊚			⊚	◦	⊚	⊚	⊚	◦	
	B-R	⊚		⊚	◦		⊚	◦	⊚	⊚	◦	⊚			◦	⊚	⊚
	R-B	◦	⊚		⊚	⊚		⊚	◦	◦		⊚	⊚	◦	⊚		⊚

*(a) In the former case, only the situations indicated by ⊚ marks were included in training data. (b) In the latter case, situations indicated not only by ⊚ marks but also by ◦ marks were included in the training data. The situations denoted as an empty cell were included in traning data in neither case. In this table, instruction patterns are abbreviated as follows. L: Left flag color; R: right flag color; A: AND-concatenated objectives; O: OR-concatenated objectives; U: up; D: down; T: true; F: false. For example, the cell referred to as DOWN-DOWN, R-G, LUF is indicated by a ⊚ mark. It means that it is possible that the robot grasping R-G flags and waiting in a DOWN-DOWN posture receives an instruction “red up false” during training in both cases of (a) and (b). As another example, the cell referred to as UP-UP, R-B, OUT is indicated by a ◦ mark. It means that it is possible that the robot grasping R-B flags and waiting in an UP-UP posture receives an instruction “red or blue up true” and “blue or red up true” during training in only the case of (b). In the other example, the cell referred to as DOWN-UP, B-R, LUT is denoted as empty. It means that the robot grasping B-R flags and waiting in an DOWN-UP posture does not receive an instruction “blue up true” during training in either case*.

**Figure 11:**
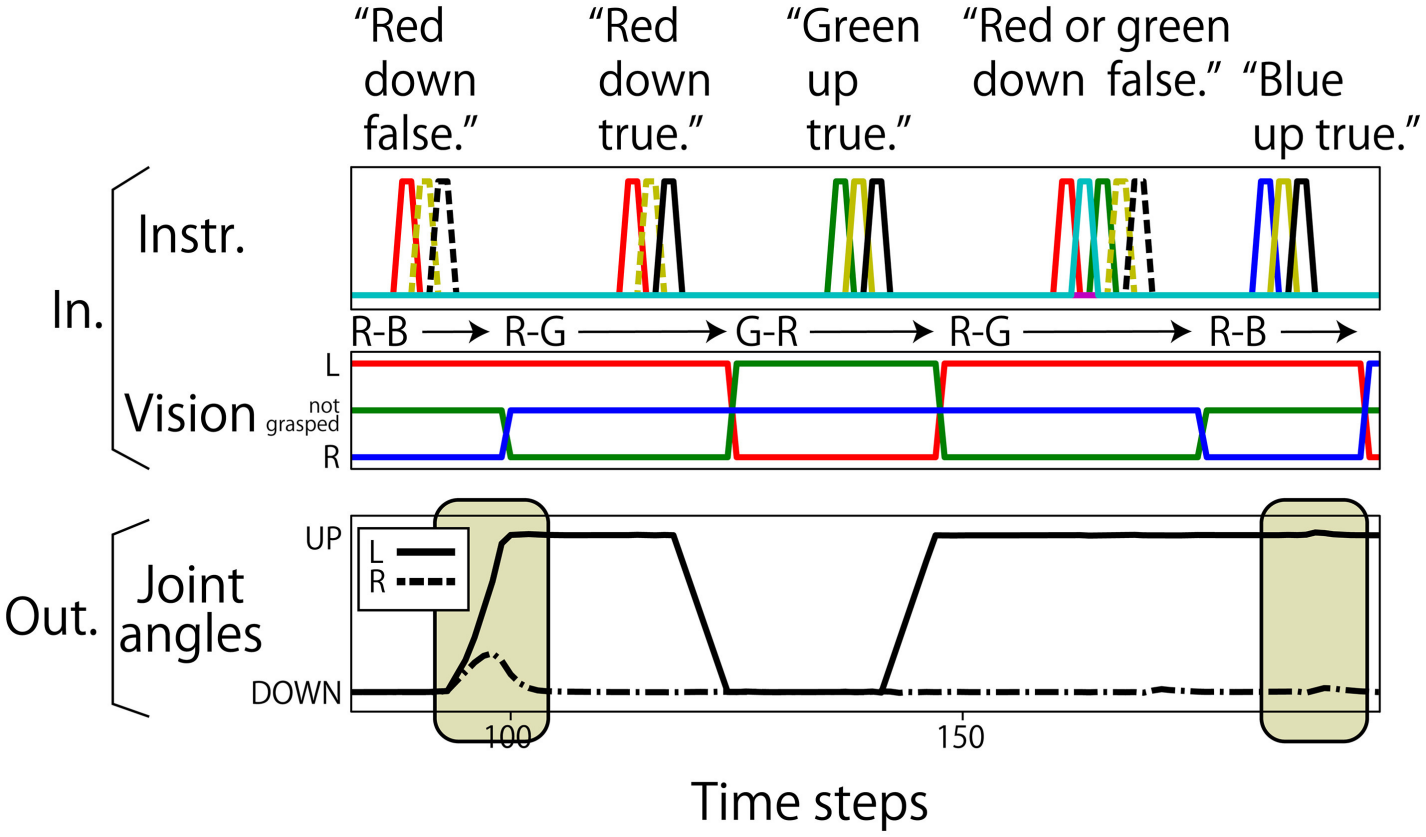
In unexperienced situations of the flag task, some different patterns of failures could be seen (indicated by beige-colored rounded boxes). The first case, indicated by the left rounded box, was that the final posture was correct but the trajectory was not stable, thus it could not satisfy the criterion that the error should be within 0.04. The second pattern was that a wrong action was chosen. In the case indicated by the right rounded box, the right arm had to be raised. However, it was actually kept in the DOWN posture.

We first explain the performance of the models trained with only 50% of possible situations. For types (2)–(4), the models behaved appropriately for many of the possible patterns, even for the unexperienced ones. In contrast, only about one-third of the possible patterns of type (1) single-objective instructions, could be dealt with appropriately. In fact, this performance matches the level from chance, in which the robot uniformly randomly chooses one of three possible motions for a single-objective instruction (moving the left arm, moving the right arm, or keeping the current posture). To clarify why the network failed to generate appropriate motions, we checked some examples actually generated by the 100-node model (Figure 11). In one failure (indicated by the left rounded box), the final posture was correct but the trajectory was not stable, and so it did not satisfy the criterion that the error should be within 0.04. In another failure (right rounded box), a wrong action was chosen. The latter case indicates that although the model roughly learned to generate some possible actions after an instruction input, it failed to learn the relationships between color words and visual information.

One possible reason for failing to respond to (1) single-objective instructions is that only this type is actually linked with visual information. For example, in the case of type (2) AND-concatenated instructions, the RNN does not have to consider visual stimuli because, when the instruction includes “and,” both arms have to be moved, regardless of the flag colors. In fact, when we tried to give the robot grasping R-B flags a contradictory instruction “green and blue up true,” it raised both arms. In other contradictory cases, the results were similar. Also for types (3) and (4), when the instruction includes “or,” either arm should be moved regardless of the flag colors. In that sense, type (1) single-objective instructions are more difficult than other types. It is possible that experiencing only half of the possible patterns is not enough to completely generalize the task space. Then, we performed the learning with the dataset in which only 25% of the situations were removed. In this case, the models responded appropriately to more than 80% of type (1) unexperienced situations in a generalized way.

In the next section, we describe another learning experiment based on the “bell task.” The bell task is different from the flag task in two ways. First, the action sequences are more complicated because we collect motion data by using a real robot. Second, all the instructions including a logic word require referring to the visual information. We investigate whether a similar kind of representations of logic words that reflect their function can be organized in more realistic setting.

## 4. Experiment 2: bell task

### 4.1. Task overview

As a more realistic task, we conducted a learning experiment based on the bell task. In contrast with the first task, we collect motion data by using a real robot. First, a human places three bells colored red, green, and blue at random: one on the left, another to the center, and the other on the right front of the robot. Then, the human gives the robot a linguistic instruction consisting of a combination of a verb (“hit,” “point”), an objective (“red,” “green,” “blue”), and an adverb (“slowly,” “quickly”). When the left or right bell is indicated, the robot must hit (point at) the bell with the closer hand. However, when the center bell is indicated, the robot can hit (point at) the bell with either hand.

Similarly to the flag task, two objective (color) words can be concatenated by “and”. In such cases, the robot has to hit (point at) the two indicated bells simultaneously. If two color words are concatenated by “or,” hitting (pointing at) either bell indicated is correct. In another case, the logic word “not” can be prefixed to a color word (referred to as NOT-prefixed). In this case, hitting (pointing at) the two bells that are the complementary set of the indicated color is the correct response. For example, when the instruction is “hit not red quickly,” the correct action is to simultaneously hit both the green and blue bells quickly.

**Figure 12:**
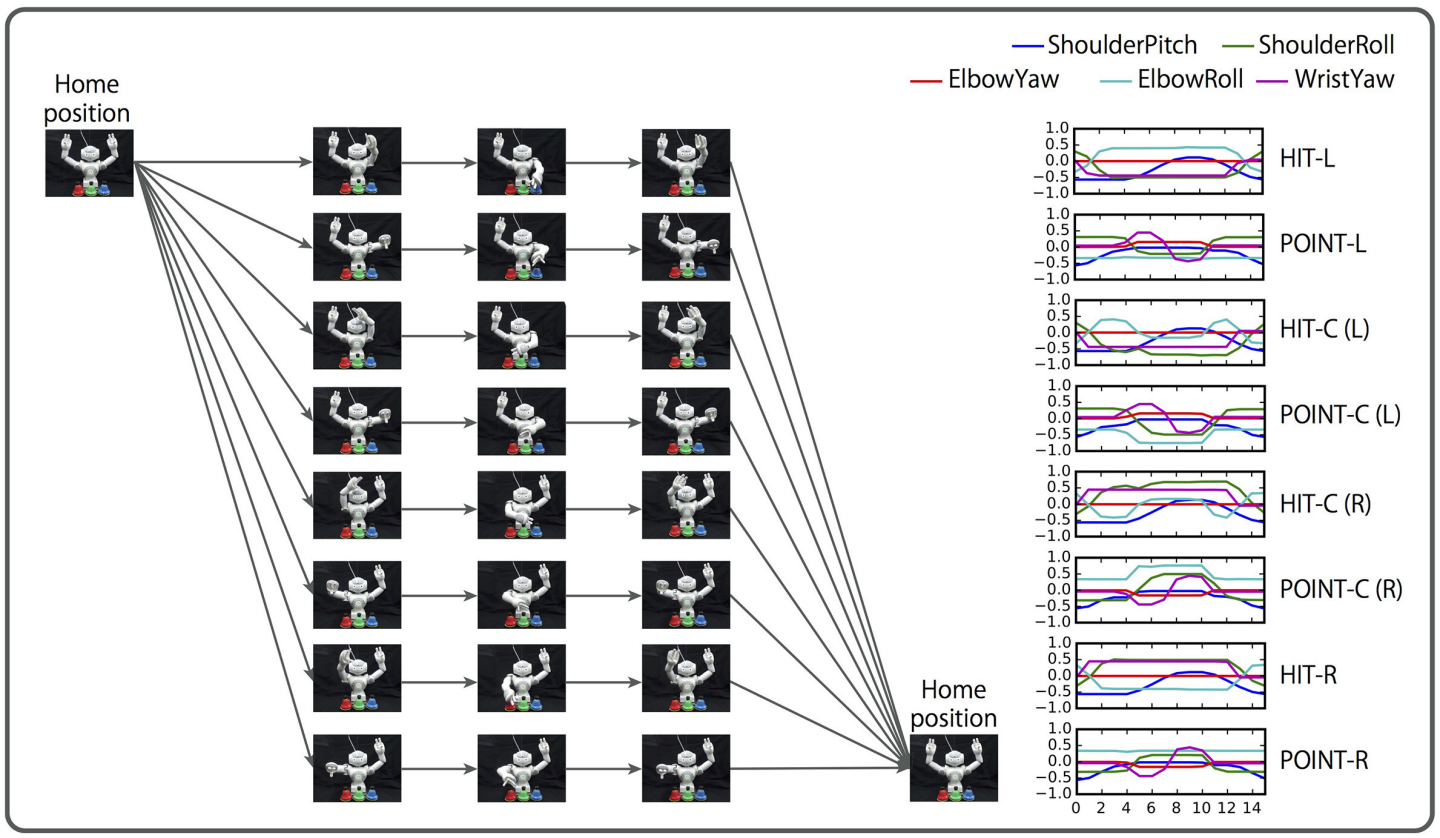
Overview of the bell task. A human places three bells colored red, green, and blue in random order. The human gives the robot an instruction consisting of a combination of a verb (“hit,” “point”), an objective (“red,” “green,” “blue”), and an adverb (“slowly,” “quickly”). When the left or right bell is indicated, the robot must hit (point at) the bell with the closer hand. In the case of the center bell, the robot may hit (point at) it with either arm. Two color words can be concatenated by “and”. In this case, the robot must act to both bells simultaneously (not presented in this figure). Two color words also can be concatenated by “or,” in which case the robot may hit (point at) either bell. In another case, the logic word “not” can be prefixed to a color word. In this case, simultaneously hitting (pointing at) the two bells that are the complementary set of the indicated color is the correct response.

The number of possible situations are 432: a combination of 72 possible instructions and 6 bell arrangements. In contrast to the flag task, in this task, the initial posture and end posture are the same, therefore the motion does not depend on the robot's initial posture. However, the actual action sequences are more complicated than the flag task, as shown in Figure 12.

### 4.2. Data representation

We represent the execution of the bell task as a sequence of 26 dimensional vectors. The state ***S***_*t*_ on time step *t* is represented as follows:

(8) $$\begin{array}{l} j_{t}=\left[j_{l0}^{\left(t\right)},j_{l1}^{\left(t\right)},j_{l2}^{\left(t\right)},j_{l3}^{\left(t\right)},j_{l4}^{\left(t\right)},j_{r0}^{\left(t\right)},j_{r1}^{\left(t\right)},j_{r2}^{\left(t\right)},j_{r3}^{\left(t\right)},j_{r4}^{\left(t\right)}\right], \end{array}$$

(9) $$\begin{array}{l} v_{t}=\left[v_{l0}^{\left(t\right)},v_{l1}^{\left(t\right)},v_{c0}^{\left(t\right)},v_{c1}^{\left(t\right)},v_{r0}^{\left(t\right)},v_{r1}^{\left(t\right)}\right], \end{array}$$

(10) $$\begin{array}{l} w_{t}=\left[w_{0}^{\left(t\right)},w_{1}^{\left(t\right)},w_{2}^{\left(t\right)},w_{3}^{\left(t\right)},w_{4}^{\left(t\right)},w_{5}^{\left(t\right)},w_{6}^{\left(t\right)},w_{7}^{\left(t\right)},w_{8}^{\left(t\right)},w_{9}^{\left(t\right)}\right], \end{array}$$

(11) $$\begin{array}{l} S_{t}=\left[j_{t};v_{t};w_{t}\right]. \end{array}$$

To represent the robot joints, 10 elements that correspond to shoulder pitch, shoulder roll, elbow roll, elbow yaw, wrist yaw on each arm are assigned to the vector ***j***_*t*_. Action sequences take approximately 16 steps in the case of QUICKLY actions, and approximately 25 steps in the case of SLOWLY actions. Action sequences are recorded by actually controlling the robot joints along predesigned trajectories. Visual information is encoded as a six-dimensional vector (***v***_*t*_). Three pairs of elements encode the bell colors. For example, *v*_*l*0_ and *v*_*l*1_ are used to represent the left bell color. In this task, it is assumed that the hues R, G, and B correspond to 0, 120, and 240° on the hue circle, respectively. The component $v_{l0}^{\left(t\right)}$ is the sine of the angle of the left bell color on the hue circle, $v_{l1}^{\left(t\right)}$ is its cosine. The pairs $v_{c0}^{\left(t\right)}$, $v_{c1}^{\left(t\right)}$ and $v_{r0}^{\left(t\right)}$, $v_{r1}^{\left(t\right)}$ encode the center and right bell colors, respectively, in the same way. This encoding method was used by Sugita and Tani (2005) and Yamada et al. (2016). Ten elements are assigned for language. Each element of ***w***_*t*_ corresponds to one word, out of “hit,” “point,” “red,” “green,” “blue,” “slowly,” “quickly,” “and,” “or,” and “not,” and an instruction sentence is represented as a sequence of 1-hot vectors. Here, the instruction sentences and corresponding action sequences are concatenated on a computer, and sequences that represent interactions are similar to those for the flag task, with multiple repetitions of instructions and corresponding actions (and waiting phases).

### 4.3. Learning setting and evaluation method

We made 512 sequential datasets for training, each of which includes eight episodes. All the possible situations were included at least once. We built models with 100, 300, 500, and 700 LSTM units, and trained them 10 times from randomly initialized learnable parameters. Adam is used as an optimizer. The number of learning iterations is 10,000, and the learning rate is set to 0.001.

After learning, we made another dataset for the evaluation which includes all possible situations 10 times. When the root mean squared errors between the generated angles and the correct ones per time step per joint during the action generations are less than 0.04, we judge that the RNN succeeds in generating an appropriate action. We regard the situation patterns in which the RNN succeeds in generating an appropriate action more than seven times out of 10 as “appropriately learned,” just as in the flag task.

### 4.4. Task performance after training

**Figure 13:**
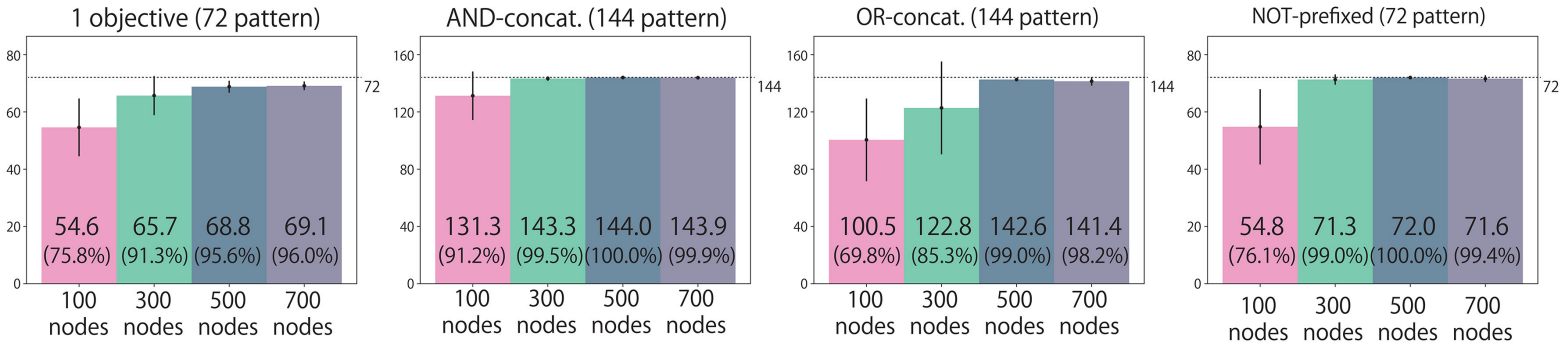
Experiment 2 (bell task). Action generation performance. We evaluated performance by counting how many situation patterns each model learned appropriately with respect to each of the four situation types: (1) the instruction includes one objective; (2) the instruction is AND-concatenated; (3) the instruction is OR-concatenated; and (4) the instruction is NOT-prefixed. The written values are averages of 10 trials in which learning began with different seeds. Error bars represent standard deviations.

We classify all the possible situations into four types: situations with (1) an instruction that includes only one objective word (72 situations); (2) an instruction is AND-concatenated (144 situations); (3) an instruction is OR-concatenated (144 situations); and (4) an instruction is NOT-prefixed (72 situations). We evaluate the performance by counting how many situation patterns each model learns appropriately with respect to each of four types. Figure 13 shows the results. The task performance was improved by increasing the number of LSTM nodes. However, there is no significant difference between 500 and 700 node models for all situation types.

Next, we investigated which action was chosen by the model for instructions that had multiple correct actions. Here, we counted the result of the model with 500 LSTM units. The situations that have multiple correct actions are divided into three types. (a) The sentence instructs the robot to act on the center bell. In this case, acting with either arm is correct; therefore, two correct actions exist. (b) The sentence instructs the robot to act on the “left or right” bell. In this case, there are also two solutions. (c) The sentence instructs the robot to act on the “left or center” bell, or the “right or center” bell. In this case, there are three answers, (i) acting on the center bell with the left arm, (ii) acting on the center bell with the right arm, and (iii) acting on the left (right) bell with the left (right) arm. The results for these three types of situation are shown in Table 2. As shown in Table 2, the model could choose each of multiple solutions evenly. In fact, types (a), (b), and (c) have 24, 48, and 96 possible variations, respectively, and the test was conducted 10 times for each of them. In most of these ambiguous situations, the RNN chose each possible solution at least once. Just as in the flag task, the RNN could learn to behave appropriately even in such ambiguous situations.

**Table 2:** Ratios of chosen solutions for ambiguous cases (two or three acceptable answers) of bell task.

**Situation**	**Choice 1 (%)**	**Choice 2 (%)**	**Choice 3**	**Failure (%)**
(a)	49.2	42.9	–	7.9
(b)	50.0	44.4	–	5.6
(c)	29.1	29.4	36.6	5.0

*The situations that have multiple correct actions are divided into three types. (a) The sentence instructs the robot to act on the center bell. In this case, acting with either arm is correct; therefore, two correct actions exist. (b) The sentence instructs the robot to act on the “left or right” bell. In this case, there are also two solutions. (c) The sentence instructs the robot to act on the “left or center” bell, or the “right or center” bell. In this case, there are three correct answers: (i) acting on the center bell with the left arm, (ii) acting on the center bell with the right arm, and (iii) acting on the left (resp., right) bell with the left (resp., right) arm*.

### 4.5. Analyses of internal representations

#### 4.5.1. Representations of “or”

**Figure 14:**
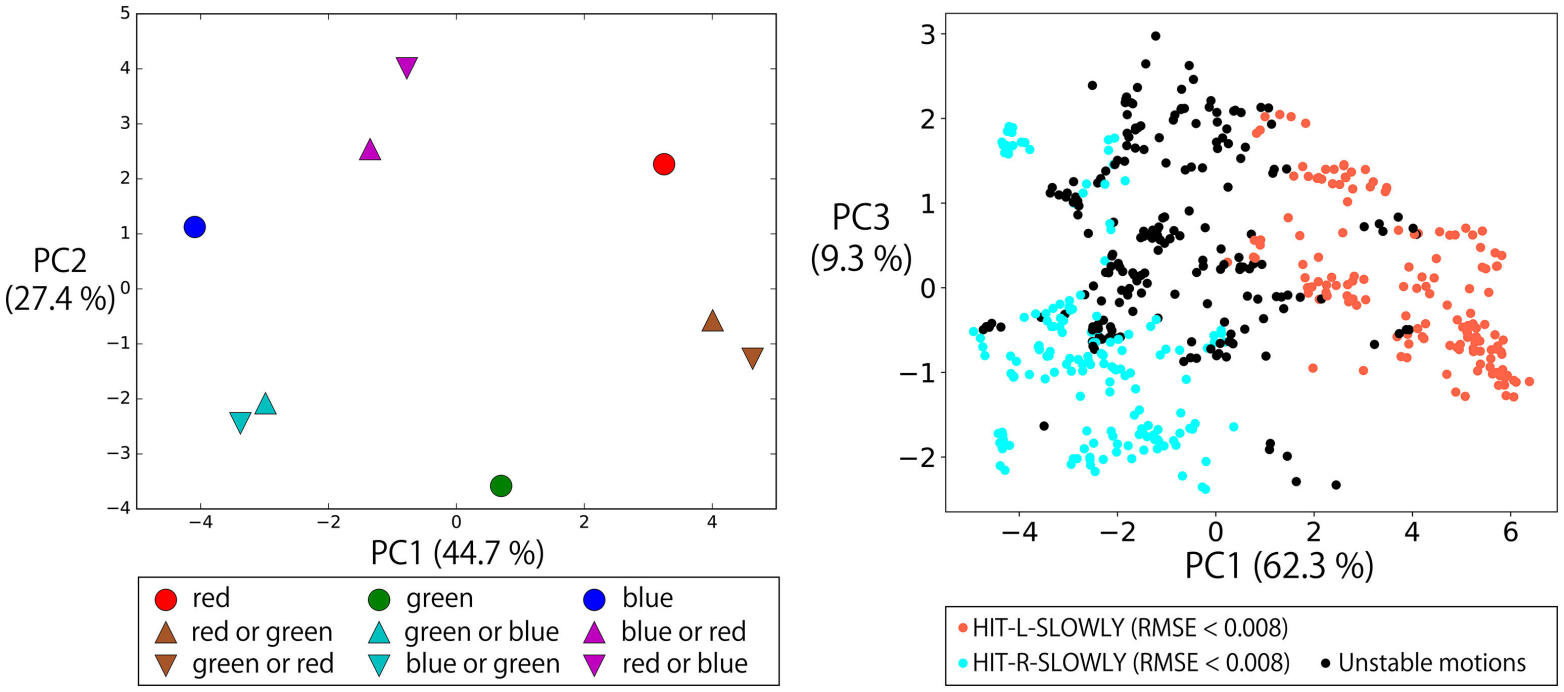
**Left**: The states of the memory cells after giving instructions in the form of “hit (objective part) slowly” that include one objective word or two OR-concatenated objective words. The activations after the OR-concatenated instructions are located between the activations for the one objective word instructions. For example, “hit red or green slowly” and “hit green or red slowly” are embedded between the encodings of “hit red slowly” and “hit green slowly.” **Right**: To a robot waiting with bells arranged in the order of RGB from left to right after 2,048 different contexts, we gave the instruction “hit red or blue slowly.” The memory cell states after the instruction were arranged on an arch shaped space which was less defined than that for the flag task. When the activation was on the left side of the arch, the robot generated HIT-L-SLOWLY action. For activation on the right side, the robot generated HIT-R-SLOWLY action. When the activation was in the topmost area, an unstable action was generated.

As in the flag task, we investigated the internal representations organized after learning by using PCA. First, we visualized the states of the memory cells after giving instructions in the form of “hit (objective part) slowly” that include one objective word or two OR-concatenated objective words (the left panel of Figure 14). This figure shows that the activations after the OR-concatenated instructions are located between the activations after the one objective word instructions. For example, “hit red or green slowly” and “hit green or red slowly” are embedded between the encodings of “hit red slowly” and “hit green slowly.” This suggests the fact that “or” is represented by unstable points in the network dynamics, as in the flag task. In fact, the right panel of Figure 14 shows an arch shaped activation space like the one in the flag task, although the shape is less clean. Note that although in the flag task, the meaning of “or” is always “left or right” regardless of the flag colors, in the current task the two candidate bells depend on the input color words and visual information. Even in this kind of situation, the functional meaning of “or” can be appropriately acquired in a way that is integrated with the objective color words.

#### 4.5.2. Representations of “and” and “not”

**Figure 15:**
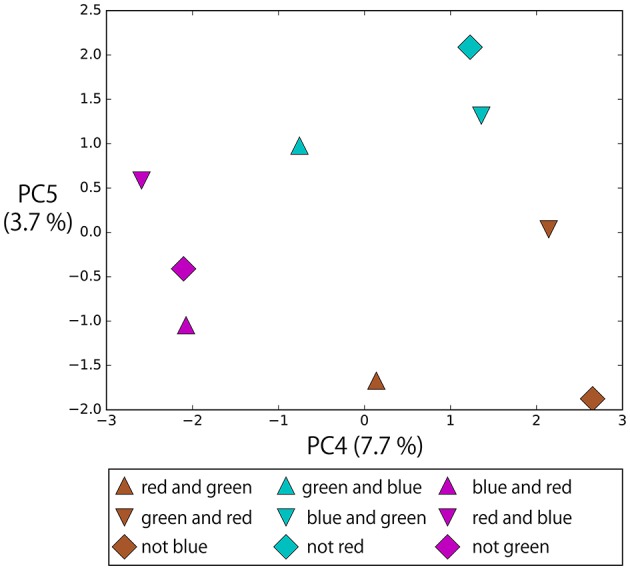
The memory cell states after giving instructions in the form of “hit (objective part) slowly,” in which objective part is AND-concatenated or NOT-prefixed. The bell arrangement was fixed in the order of R, G, B from left to right. In this task, “not” indicates the complementary set. Therefore, for example, “not green” and “red and blue” have the same meaning. Although the objective parts of these instructions are completely orthogonal, they are located close each other in the space spanned by PC4 and PC5 and, as a result, instructions with the same meaning form clusters: R-AND-G, G-AND-B, and B-AND-R.

Figure 15 shows the memory cell states after giving instructions in the form of “hit (objective part) slowly,” in which the objective part is AND-concatenated or NOT-prefixed. The bell arrangement was fixed in the order of R,G,B from left to right. In this task, “not” indicates the complementary set. Therefore, for example, “not green” and “red and blue” have the same meaning. Although the objective parts of these instructions are completely orthogonal to each other, they are located close each other in the space spanned by PC4 and PC5 and, as a result, instructions with the same meaning form clusters: that is, R-AND-G, G-AND-B, and B-AND-R. These instructions including logic words also require the RNN to consider visual information to determine the meaning of the sentence. Which two bells should be hit (pointed at) depends on both the input color words and visual information. The RNN learned to link these sentences flexibly in the sensorimotor information just from the experience of sequential data for the imposed task.

In summary, even in the bell task that requires both referring to visual information and processing of logic words simultaneously, the functional meaning of logic words could be appropriately organized in a way that was integrated with the referential words.

## 5. Discussion

The current study conducted learning experiments involving translation from linguistic instructions, including both referential and logic words, into robot actions in order to investigate what kind of compositional representations emerged from the interactive experiences. In the case of referential words, objective words were merged with visual input, verbs were integrated with the robot's own posture, and as a result, appropriate actions were generated. Simultaneously, the model could also deal with the logic words “true,” “false,” “not,” “and” and “or”. By embedding these words as internal representations that reflect their functional properties, appropriate actions were achieved. In this following, we discuss three types of logic word separately.

### 5.1. True, false, not

“True” and “false” in the flag task were understood as the goal-oriented action UP/DOWN by being combined with “up” and “down” in a X-OR manner. “Not” in the bell hitting task worked as an operation to choose a complementary set. For example, “not red” corresponded to “green and blue.” The RNN learned to embed these completely orthogonal phrases as having the same meaning in the lower-ranking principal component space by its non-linear transformation. In the field of natural language understanding by deep learning, a similar kind of analysis has been performed. Li et al. (2016) showed that a model optimized for sentiment analysis changes its internal encoding drastically in response to the negation of an expression. Hence, for example, “not good” is encoded closer to “bad” than to “good”. However, the visualization in the current study showed that even though the information that input sentences were completely different is still retained in the main component space, the combined representation corresponding to the behavioral meaning is reflected in the lower ranking principal components. In other words, not only information encoding compositionally integrated meaning but also information of compositional elements are retained in the model's memory.

This aspect seems to be important. For example, imagine that both of the sentences “hit red quickly” in the case of an RGB bell arrangement and “hit blue quickly” in the case of a BGR arrangement were encoded just as the action HIT-L-QUICKLY with the loss of the information about element words. In this case, it would be impossible for the model to respond appropriately to changes, such as a sudden replacement of bells during the action generation, because the color word information has been lost. By retaining the information about compositional elements, adaptive behavior to respond to such fluctuations would be possible, although it is not certain that our current model is capable of dealing with such situations because they were not included in training data.

### 5.2. And

In the flag task, “and” *per se* worked as a kind of universal quantifier without referring to objective words. For example, when a robot grasping R-B flags was given “green and blue up true,” it lifted up both arms. In other contradictory cases, the results were similar. In other words, if the instruction includes “and,” the color words are ignored and only the verb (and truth value) is considered. In that sense, “and” is represented as a concept one step higher. This interpretation of “and” by the neural network could not be expected before the experiment and is actually out of our common usage of “and”; but it can be seen as a reasonable and rational solution in the range of the current task. In contrast, in the bell task, AND-concatenated instructions required referring to visual information, and the model appropriately integrated them with the visual information and then generated correct both-hand actions.

In this way, “and” was represented in a different suitable manner with respect to each task. However, in general, there are more situations in which “and” is used in different ways to combine words, phrases, or sentences. The investigation of how such higher order or general types of “and” can be handled or represented is left for future work.

### 5.3. Or

In the flag task it was shown that without noise applied to the joint angles, the model learned less successfully than it did with noise. This difference did not appear in preliminary experiments that did not include OR-concatenated instructions. We think that due to the inclusion of OR-concatenated instructions that introduce ambiguity by giving as correct either of the answers randomly each time, the optimization by minimization of the simple squared error became unstable. This is a very similar to a popular thought experiment called Buridan's ass. In the story, an ass is given grass feed on both its left and right sides, located at exactly the same distance away. Faced with this dilemma it could not choose a side and finally starved to death. Our analysis shows the possibility that the network solved this problem, which the ass faced too honestly, by using the tiny amount of noise as a clue to determine which arm moves and by organizing unstable dynamics which converges to either of two fixed-point attractors. However, a more detailed analysis of the dynamical characteristics of the model is required. For example, Tani and Fukumura (1995) showed that a deterministic RNN model can reproduce a simple symbol sequence that is generated in accordance with probabilistic rules by producing a self-organizing chaotic dynamics. Namikawa et al. (2011) also demonstrated that a temporally hierarchical RNN could learn to generate pseudo-stochastic transitions between multiple motor primitives on a robot. Our experiment showed that a similar kind of function to generate actions as if they were generated probabilistically is achieved from the learning of an interactive instruction-action task that includes longer time dependency and more complexity. Our results also showed that the ability to deal with OR-concatenated instructions was improved by increasing LSTM node numbers. We think that by increasing the number of nodes and improving the representation ability the network could learn to forcibly embed the probabilistic experiences in a chaotic dynamics. We should analyze how the function is dynamically represented in the future.

### 5.4. Summary and future work

This study conducted learning experiments that translates linguistic sentences, including both referential and logic words, into robot actions to investigate what kind of compositional structures emerged from the experiences of interaction. Referential words were linked in the visual information and the robot's current state and then appropriate actions were generated. The logical words were also simultaneously represented by the model in accordance with their functions as logical operators. To be more precise, the words “true,” “false” and “not” work as non-linear transformations to embed orthogonal phrases into the same area in a lower-rank principal component space. “And” in the flag task eliminated referring to the visual information in a rational way and worked as if it *per se* was a universal quantifier. “Or,” which requires action generation that looks apparently random, was represented as an unstable space of the network's dynamical system.

Future work includes the following. First, we should confirm whether both referential and logic words are simultaneously learned when the complexity of the task is more extended. Although the scaling up of vocabulary size is one way to extend, the scaling up of syntactic variety is also required because the sentence patterns in this study were fixed in each task. In extended tasks, it would be possible that the logic words are used not only between words but also between phrases or clauses. Moreover, although the visual information in the current experiments is highly preprocessed, in more realistic tasks, the environment would include various meaningful information, not only color. Therefore, the relationships between language and the environment should be learned from low-level data (e.g., raw images) in a less arbitrary way. To deal with such tasks, we could extend our model by replacing the preprocessing module with another neural network model for vision, such as a convolutional neural network (CNN). In fact, some studies have actually combined a CNN with an RNN to learn the relationships between linguistic instructions and corresponding behavior in an end-to-end manner (Chaplot et al., 2017; Hermann et al., 2017).

Second, a more detailed analysis of the internal representations is required. This includes the analysis of more dynamical characteristics and the visualization of the activation patterns of each neuron. In particular, the latter seems to be valuable, because, although in the current study we visualized activation only in the principal component space, models that have memory cells, such as gated recurrent units or LSTM, are expected to encode different information and functions in specific nodes.

Finally, we are planning to build a bi-directional neural model to translate between linguistic and behavioral sequences. In fact, human language systems are bi-directionally translatable. To build a bi-directional model would be valuable both for understanding symbol grounding structure more deeply and for developing more flexible communicative agents.

## Author contributions

TY, SM, HA, and TO conceived and designed the research, and wrote the paper. TY performed the experiment and analyzed the data.

### Conflict of interest statement

The authors declare that the research was conducted in the absence of any commercial or financial relationships that could be construed as a potential conflict of interest.

## Footnotes

^1^In this paper, we use the term “referential” instead of “grounded” for the following reason. We conduct two robot experiments in the following sections, but the first task is numerically simulated on a computer. Even though the second task uses a real robot, the visual input is still highly preprocessed. Strictly speaking, we do not deal with the symbol grounding problem in accordance with the definition by Harnad (1990). To prevent misunderstanding, we use the term “referential,” and sometimes “linking” to express that a word has a referent or a corresponding feature in other sensorimotor modalities.

^2^In this paper, we denote specific actions or behaviors executed by agents with capital letters.

^3^Before applying PCA, parallel translation was applied to the internal state vectors to make the mean of them the zero vector (i.e., centering preprocessing was performed).
